# Repurposing a detrimental antibody epitope as targeted therapeutics for sepsis and rheumatoid arthritis

**DOI:** 10.1186/s40779-026-00686-8

**Published:** 2026-02-17

**Authors:** Wei-Qiang Chen, Li Lou, Xiao-Ling Qiang, Cassie Shu Zhu, Jian-Hua Li, Shu-Jin Chen, Brian Xiong, Huan Yang, Ping Wang, Kevin J. Tracey, Hai-Chao Wang

**Affiliations:** 1 The Feinstein Institutes for Medical Research, Northwell Health, Manhasset, NY 11030 USA; 2 Departments of Emergency Medicine and/Or Molecular Medicine, Donald and Barbara Zucker School of Medicine at Hofstra/Northwell, Hempstead, NY 11549 USA

**Keywords:** High mobility group box 1 (HMGB1), Procathepsin L, Tetranectin, Sepsis, Rheumatoid arthritis (RA), Antibody, Mimetic peptide, Epitope, Innate immune cells

## Abstract

**Background:**

Sepsis and rheumatoid arthritis (RA) are distinct yet mechanistically related conditions commonly driven by dysregulated inflammatory responses. Here, we explored the counterintuitive hypothesis that an epitope from a deleterious anti-tetranectin (TN) antibody (mAb9) could hold unforeseen therapeutic potential.

**Methods:**

By mapping mAb9’s epitope to P2 (residues 55–70), a region crucial for TN’s protective functions, we developed P2-1, a water-soluble derivative as a targeted therapy. We then employed animal models of sepsis (cecal ligation and puncture) and arthritis (collagen antibody-induced arthritis) to evaluate the therapeutic effects of P2, P2-1, and a procathepsin L (pCTS-L)-neutralizing antibody by assessing septic survival, arthritis severity, pain sensitivity, and joint tissue histology. In parallel, we utilized a surface plasmon resonance (SPR) assay and computational modeling to examine the P2-1/high mobility group box 1 (HMGB1) interaction. Finally, we elucidate the effect of P2-1 on the HMGB1-induced release of pCTS-L and other cytokines and chemokines using primary human peripheral blood mononuclear cells (PBMCs).

**Results:**

P2-1 significantly improved survival and reduced systemic inflammation in a sepsis model, and attenuated arthritis severity and pain sensitivity in an RA model, even with therapeutic administration after disease onset. Mechanistically, P2-1 exhibited high-affinity binding to HMGB1 and selectively suppressed HMGB1-induced cathepsin L (*Ctsl*) mRNA upregulation and pCTS-L secretion from human immune cells, crucially without perturbing other HMGB1-induced cytokines and chemokines. We further validated pCTS-L as a therapeutic target by demonstrating that a neutralizing antibody conferred potent antiarthritic effects, reducing joint inflammation, pain, and structural damage.

**Conclusions:**

Our findings introduce a paradigm-shifting drug discovery strategy that transforms insights from harmful antibody action into targeted therapeutics for the HMGB1-pCTS-L axis. This approach not only delivers P2-1 as a potent therapy but also establishes pCTS-L as a crucial mediator in inflammatory diseases such as sepsis and RA.

**Supplementary Information:**

The online version contains supplementary material available at 10.1186/s40779-026-00686-8.

## Background

Sepsis, a life-threatening acute inflammatory syndrome, accounts for nearly 20% of deaths worldwide [1] and imposes a substantial economic burden that exceeds $62 billion annually in the USA alone. Its complex pathogenesis is partly driven by dysregulated innate immune responses, which are initiated by early proinflammatory cytokines [e.g., tumor necrosis factor (TNF)] [2] and subsequently sustained by late-acting damage-associated molecular patterns (DAMPs) such as high mobility group box 1 (HMGB1) [3, 4] and inducible procathepsin L (pCTS-L) [5, 6]. In contrast, rheumatoid arthritis (RA) is a chronic autoimmune disease that affects 0.5–1.0% of the global population [7] and is characterized by persistent synovial inflammation and progressive joint destruction. Despite their distinct etiologies and clinical manifestations, both conditions converge on a common immunopathological mechanism: the dysregulated release of key proinflammatory mediators, including cytokines [e.g., TNF, interleukin (IL)-1, and IL-6] and DAMPs such as HMGB1 [3, 4, 8–10]. Although numerous clinical trials have failed in sepsis, preclinical insights into pathogenic cytokines have facilitated the development of highly effective anti-TNF monoclonal antibodies (mAbs) such as infliximab, etanercept, and adalimumab, which now serve as cornerstone therapies for RA [11–13].

Our previous work, spanning two decades, established HMGB1 as a pivotal inflammatory mediator in both lethal sepsis [3, 4] and RA [9, 10]. Upon binding to cell surface receptors such as Toll-like receptor 4 [14, 15] or the receptor for advanced glycation end products (RAGE) [16], extracellular HMGB1 either induces cytokines/chemokines or, if internalized through RAGE-mediated endocytosis, triggers macrophage pyroptosis [17, 18] (Additional file 1: Fig. S1). As a highly charged protein, HMGB1 interacts with other endogenous proteins, including haptoglobin [19], complement component 1q [20], and tetranectin (TN) [21], thereby triggering anti-inflammatory responses via distinct signaling pathways (Additional file 1: Fig. S1). In contrast, cathepsin L (*Ctsl*) is highly inducible in monocytes/macrophages and synovial fibroblasts by bacterial endotoxins [e.g., lipopolysaccharides (LPS)] and endogenous cytokines [e.g., interferon-γ, IL-6, and serum amyloid A (SAA)] [5, 22, 23]. Its precursor, pCTS-L, can be secreted extracellularly by activated innate immune cells, functioning as a late-acting mediator in lethal sepsis [5, 6, 24–26]. Consequently, pCTS-L levels are significantly elevated in the serum and/or synovial fluid of patients with sepsis [21] or RA [27–29]. In RA animal models, *Ctsl* mRNA and CTS-L protein levels are elevated in synovial tissues, particularly within the sublining layer and perivascular infiltrates (e.g., macrophages) [30]. These CTS-L-rich macrophages may directly contribute to subchondral bone erosion, a hallmark of chronic RA [31], as genetic depletion or pharmacological suppression of *Ctsl* expression attenuated antigen-induced arthritis [32] and cartilage destruction in mice [33]. However, it is currently unknown whether HMGB1 can induce pCTS-L expression and secretion in human immune cells, or whether pharmacological suppression of pCTS-L expression or extracellular activity could offer protection against sepsis and RA in preclinical settings.

Physiologically, TN circulates abundantly (8–12 µg/ml) in healthy individuals [34], but its levels decrease markedly in patients with sepsis [21], heart failure [35, 36], or RA [37]. Human TN comprises a heparin-binding region [38], an oligomerization α-helical segment, and a carbohydrate recognition domain (CRD). The CRD facilitates binding to multiple proteins, including plasminogen [39], tissue-type plasminogen activator [40], and HMGB1 [21]. Specifically, detrimental TN/HMGB1 interactions promote cellular uptake (Additional file 1: Fig. S1), leading to macrophage pyroptosis and immunosuppression, which may compromise microbial eradication [21]. Intriguingly, some TN domain-specific mAbs reduce septic lethality [21, 41], whereas others that target a distinct epitope paradoxically increase it [21]. This led us to hypothesize that the epitope of a detrimental anti-TN mAb could be inversely developed as a therapy for inflammatory diseases such as sepsis and RA.

Despite therapeutic advances, novel sepsis and RA therapies are urgently needed, as both diseases involve diverse immune pathways and heterogeneous patient populations [8]. Although anti-TNF therapies have revolutionized RA treatment [11, 12], they offer only partial efficacy for some patients [42] and can adversely increase susceptibility to infections [43]. Patients may also develop tolerance [44] or anti-drug antibodies over time [45], progressively diminishing the long-term effectiveness of these therapies. Given these limitations, we explored the therapeutic efficacy of an innovative TN-derived P2-1 peptide and a pCTS-L-neutralizing mAb. Here, we present compelling evidence for the therapeutic potential of a TN-derived P2-1 peptide and a pCTS-L-neutralizing monoclonal antibody (mAb2) in animal models of two inflammatory diseases.

## Methods

### Animal models and ethics statement

We adhered to the ARRIVE guidelines for minimizing animal numbers and the Minimum Quality Threshold in Preclinical Sepsis Studies [46]. Practices included randomization, delayed therapeutic interventions [47], the establishment of criteria for euthanizing moribund animals (e.g., labored breathing, minimal response to touch, and immobility), and the administration of fluid resuscitation and antibiotics to septic animals. This study was approved by the Institutional Animal Care and Use Committee (IACUC) of the Feinstein Institutes for Medical Research (Protocol #2017–027 Term II, August 24, 2020; #2023–002, April 6, 2023; and #2024–0846, October 29, 2024). Male and female BALB/c mice (8–10 weeks old, 20–25 g) were obtained from Jackson Laboratory (Bar Harbor, ME, USA) or Charles River Laboratories (Wilmington, MA, USA), and acclimated for at least 5–7 d under specific pathogen-free conditions with free access to food and water before experimentation. Experiments were conducted in sex-segregated groups to assess potential sex-specific differences; data were pooled when no significant differences were observed, thereby minimizing the number of animals used while ensuring robust conclusions. Calculating the number of animals needed for an experiment using power analysis is a critical step in ethical and efficient research. It ensures that our study was adequately powered to detect a biologically meaningful effect, avoiding the use of too few animals (leading to inconclusive results and wasted resources) or too many animals (which is unethical). As stated in our approved IACUC protocols, we performed power analysis for each experiment based on the estimated standard deviation of our primary outcome measure. This information was derived either from our own robust pilot studies or from relevant, peer-reviewed previous literature using similar animal models and experimental conditions. This approach allowed us to determine the minimum sample size required to detect an anticipated biologically significant effect size with a specified level of statistical power, while controlling for Type I error.

### Cecal ligation and puncture (CLP) sepsis model

Experimental sepsis was induced in BALB/c mice via CLP as previously described [5, 21, 24]. Before CLP surgery, all the animals received buprenorphine once (0.05 mg/kg, subcutaneously) for pain management, as repetitive buprenorphine use in CLP could paradoxically elevate sepsis surrogate markers and increase animal lethality [48, 49]. The animals were randomly assigned to the control vehicle (*n* = 10 or *n* = 20) or experimental groups (*n* = 10 or *n* = 20). P2 (5.0 mg/kg), P2-1 (0.1 mg/kg or 1.0 mg/kg), or tetranectin N-terminal deletion mutant (ΔTN) (1.0 mg/kg) were administered intraperitoneally (i.p.) at the indicated time points, and survival rates were monitored. To elucidate P2-1’s protective mechanisms, a separate group of BALB/c mice (*n* = 4) underwent CLP and received P2-1 (1.0 mg/kg) at 2 h and 20 h post-CLP. At 24 h post-CLP, the animals were euthanized, blood was collected, and serum cytokines and chemokines were measured via murine cytokine antibody arrays as previously described [21]. More details are presented in Additional file 1: Methods.

### Collagen antibody-induced arthritis (CAIA) model

The CAIA model was established in BALB/c mice (8–10 weeks old) as described previously [10]. On Day 0, the mice received an i.p. injection of 2 mg of anti-collagen II mAb cocktail (α-CII, Chondrex, Inc., Redmond, WA, USA). On Day 3, the mice received an i.p. injection of 30 µg of LPS [*Escherichia coli* (*E. coli*) O111:B4; Sigma-Aldrich, St. Louis, MO, USA] to activate inflammation and synchronize arthritis onset. The mice were randomized to the control vehicle (*n* = 5 or *n* = 7–10) or experimental groups (*n* = 5 or *n* = 7–10). Therapeutic agents [P2-1 peptide (1.0 or 2.0 mg/kg) or pCTS-L-neutralizing mAb2 (1.0, 2.0, or 4.0 mg/kg)] were administered i.p. daily, either prophylactically from–Day 2 or therapeutically from Day 6 post-α-CII challenge. Arthritis severity was scored daily via a clinical scoring system [0–4 per paw: 0 = normal; 1 = mild redness and swelling in a single joint type (ankle, wrist, or individual digit); 2 = moderate redness and swelling affecting two joint types; 3 = severe redness and swelling across all 3 joint types; 4 = maximal redness and swelling of the entire paw that obscured the joint definition].

### Pain sensitivity assessment

Mechanical allodynia was assessed using Von Frey filaments (Stoelting Co., Wood Dale, IL, USA) applied to the plantar surface of the hind paws as previously described [10]. The mice (*n* = 6–10) were habituated to individual transparent enclosures on an elevated wire mesh platform. Calibrated Von Frey filaments (0.008–2.000 g) were applied for 2–3 s or until a withdrawal response occurred. The “up-down” method determines the 50% paw withdrawal threshold, defined as the force required to elicit a withdrawal response in 50% of applications.

### Histopathological analysis

Ankle joints were harvested from CAIA mice (*n* = 10) at the end of the study, decalcified in 10% ethylenediaminetetraacetic acid/sucrose (Cat. #1048B, Newcomer Supply, Waunakee, WI, USA), and embedded in paraffin. Sections (5 µm) were cut and stained with hematoxylin and eosin to assess overall morphology and inflammation. As previously described [50], histopathological scoring was performed by a blinded pathologist based on established criteria: synovial inflammation (0–3 scale: 0 = no inflammation, 1 = mild, 2 = moderate, 3 = severe), bone erosion (0–3 scale: 0 = no erosion, 1 = mild, 2 = moderate, 3 = severe), and cartilage erosion (0–3 scale: 0 = no erosion, 1 = mild, 2 = moderate, 3 = severe). Representative images were captured via a brightfield microscope (Nikon Eclipse Ti2, Melville, NY, USA).

### Protein expression, purification, and peptide synthesis

Recombinant rat HMGB1, a 33 kD fusion protein with an N-terminal calmodulin-binding peptide (3 kD) tag, was expressed in *E. coli* BL21 (DE3) pLysS cells after its cDNA was cloned and inserted into a pCAL-n vector as previously described [3, 51]. A recombinant ΔTN, lacking residues 1–44 (heparin binding and part of the α-helix trimerization domain), was expressed in DE3 pLysS with an N-histidine tag, and was purified to homogeneity following a similar protocol to that for full-length TN [21]. The P2 peptide (55-KVHMKCFLAFQTKTF-70; Lot # U114VHD110-1/PE6854; Purity, 98.1%) and its water-soluble derivative P2-1 [54-TKVH(Nle)KSFLAFQTKT-69; Lot # U114VHD110-4/PE6857; Purity, 99.6%] were custom-synthesized (GenScript, Piscataway, NJ). The pCTS-L-neutralizing mAbs (mAb2) and TN-neutralizing mAbs (mAb8 and mAb9) were generated in BALB/c and C57BL/6 mice as previously described [5, 21].

### Surface plasmon resonance (SPR) assay

Binding kinetics between HMGB1 and P2-1 were measured via Nicoya Life Science gold nanoparticle-based OpenSPR technology (Kitchener, ON, Canada) as previously described [5, 52]. Recombinant human HMGB1 was immobilized onto a carboxyl sensor chip, and the P2-1 peptide was flowed over the immobilized HMGB1 at various concentrations in HEPES-buffered saline with poloxamer (HBS-P buffer, GE Healthcare, Chicago, IL, USA). The response units were recorded over time, and binding affinity was estimated as the equilibrium dissociation constant *K*_D_ using Trace Drawer Kinetic Data Analysis v.1.6.1 (Nicoya Life Science). Given the 1:1 stoichiometry of the HMGB1-P2-1 interaction predicted by ClusPro protein-protein docking (Additional file 1: Fig. S2), we applied a 1:1 interaction algorithm to determine the association rate constant (k_a_), dissociation rate constant (k_d_), and equilibrium dissociation constant (K_D_) from three independent experiments (*n* = 3 technical replicates).

### P2 peptide structure prediction and ClusPro protein-protein docking

To predict the 3D structure of the P2 peptide, its amino acid sequence was submitted in FASTA format to the Iterative Threading ASSembly Refinement (I-TASSER) web server (https://zhanggroup.org/I-TASSER/). I-TASSER predicts peptide 3D structures by integrating template-based threading, ab initio modeling, and molecular dynamics simulations to construct and refine models [53]. It generates several predicted models, each accompanied by a C-score ranging from −5 to 2, where higher values indicate higher confidence and better model quality. To elucidate P2/HMGB1 interaction details, the binding interface between the HMGB1 B-box and P2 was predicted via the ClusPro Web Server (https://cluspro.org). The crystal structure of HMGB1 B-box (PDB: 1HME) was used as the receptor, and the I-TASSER predicted structure of the P2 peptide (Additional file 1: Fig. S3 a, Model 1; Additional file 2) was used as the ligand. The docking algorithm explored various binding modes, and the complex with the lowest Gibbs free energy was selected for visualization and analysis of potential interaction sites.

### Human peripheral blood mononuclear cell (PBMC) isolation and culture

Human blood was purchased from the New York Blood Center (Long Island City, NY, USA), and PBMCs were isolated via density gradient centrifugation through Ficoll (Ficoll-Paque PLUS) as previously described [5, 21]. PBMCs were cultured in RPMI-1640 supplemented with 10% human serum, 2 mmol/L L-glutamine, 100 U/ml penicillin, and 100 µg/ml streptomycin (all from Invitrogen, Carlsbad, CA, USA). For experiments, PBMCs were stimulated with recombinant HMGB1 (0.5 or 2.0 µg/ml) for 16 h, either alone or in combination with P2-1 at 5.0 or 10.0 µg/ml. Cell-free supernatants were collected for analysis of cytokine/chemokine levels via cytokine antibody arrays, or pCTS-L levels via Western blotting analysis as previously described [5].

### RNA sequencing (RNA-Seq) and bioinformatics analysis

Total RNA was isolated from human PBMCs (*n* = 6 biological replicates) stimulated with HMGB1 in the absence or presence of P2-1 (5.0 or 10.0 µg/ml) via the RNeasy Mini Kit (Qiagen, Germantown, MD, USA) according to the manufacturer’s instructions. cDNA libraries were prepared using the TruSeq RNA Library Prep Kit v2 (Illumina, San Diego, CA, USA) and sequenced on an Illumina NovaSeq 6000 platform to generate 100 bp paired-end reads. Gene counts were quantified via StringTie and DESeq2 for differential gene expression analysis. Volcano plots and heatmaps were generated via ggplot2 and pheatmap packages in R, respectively. Genes with an adjusted *P*-value < 0.05 and a |log_2_ fold change|> 1 were considered differentially expressed.

### Cytokine antibody array analysis

The levels of cytokines and chemokines in mouse serum and joint tissue lysates, or human PBMC culture supernatants, were determined via murine or human cytokine antibody arrays (e.g., Cat. #AAM-CYM-3–8 or Cat. #AAH-CYT-3–8, RayBiotech Inc., Norcross, GA, USA) as previously described [5, 21, 54, 55]. For the sepsis model, blood samples were collected from the mice (*n* = 4) at 24 h post-CLP. For the CAIA model, soft tissues from the hindpaw and ankle joints were carefully harvested from normal (*n* = 2) and CAIA (*n* = 4) mice using a previously established protocol [56]. Briefly, 0.5 g of fresh soft tissue was cut into 3 mm pieces and mixed with 400 µl of a hypertonic solution (500 ml of PBS with 4.5 g of NaCl and 2 × protease inhibitor cocktail) in a 10 ml syringe. Mechanical extrusion, involving 30 rapid back-and-forth movements of the plunger, was then performed. The expelled extracellular fluid was collected, immediately aliquoted, and stored at −20 °C until analysis. Cell-conditioned medium was collected from PBMC cultures after 16 h of stimulation. Arrays were performed according to the manufacturer’s protocol, followed by chemiluminescent detection and densitometric quantification as previously described [5, 21].

### Western blotting analysis of pCTS-L

Cell-conditioned medium from human PBMC cultures was collected and concentrated via Amicon Ultra Centrifugal Filters with a 10 kD molecular weight cutoff (Cat. #UFC801096, MilliporeSigma, Burlington, MA, USA). Equal volumes of conditioned medium, representing equivalent numbers of cells, were resolved on sodium dodecyl sulfate-polyacrylamide gels and transferred to polyvinylidene difluoride membranes. After being blocked with 5% nonfat milk in Tris-buffered saline with 0.1% Tween 20, the membranes were probed with a homemade primary monoclonal antibody against pCTS-L (e.g., mAb2, 1:1000) overnight as previously described [5]. After washing, the membranes were incubated with a horseradish peroxidase-conjugated secondary antibody. The protein bands were visualized using enhanced chemiluminescence substrate (Pierce) and detected via a chemiluminescence imaging system (e.g., Bio-Rad ChemiDoc MP). The relative levels of specific proteins were determined using the UN-SCAN-IT gel analysis software version 7.1 (Silk Scientific Inc., Orem, UT, USA) and expressed in arbitrary units (AU). The experiments were performed in quadruplicate (*n* = 4 biological replicates).

### Statistical analysis

All the data were first assessed for normality via the Shapiro-Wilk test before the appropriate statistical analyses were performed. All the data are presented as the mean ± standard errors of the means unless otherwise specified. For comparisons among multiple groups with nonnormal (skewed) distribution, the Kruskal-Wallis ANOVA test followed by Dunn’s post-hoc test was used to evaluate significant differences. Survival curves were analyzed via the Kaplan-Meier method and compared via the nonparametric log-rank (Mantel-Cox) test. Statistical significance was defined as a *P*-value less than 0.05.

## Results

### Mapping the epitope of a detrimental anti-TN antibody

**Fig. 1:**
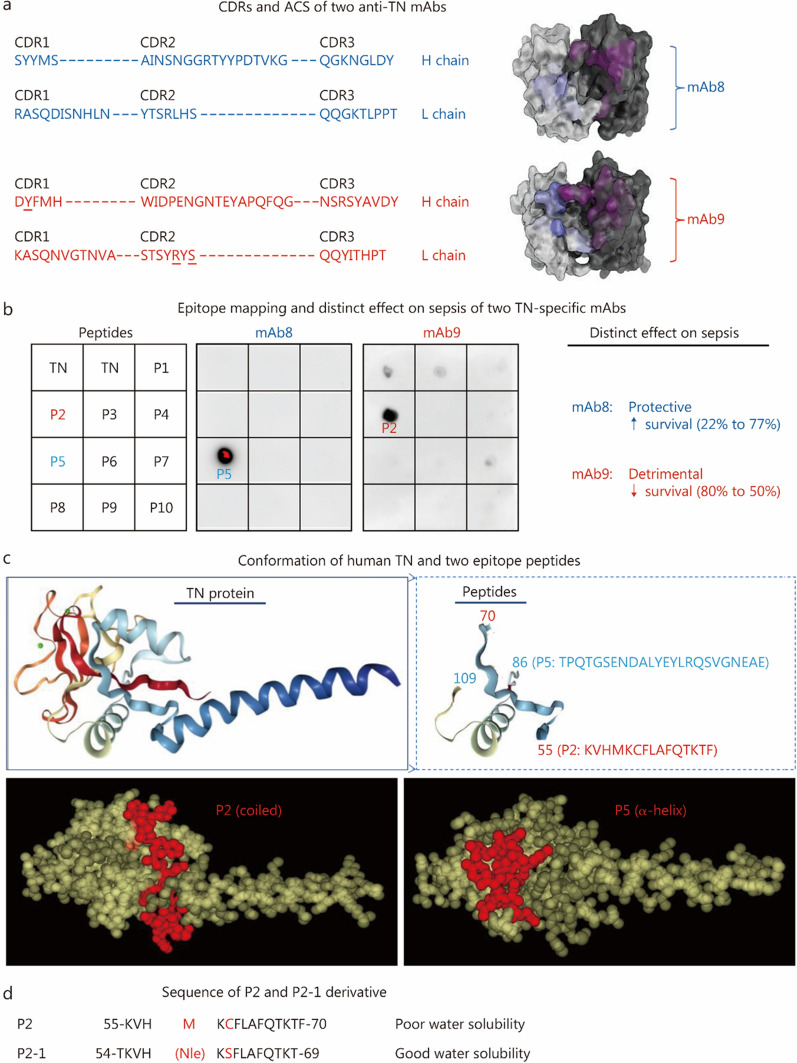
Identification and engineering of P2-1 as an epitope peptide from the detrimental anti-TN mAb (mAb9). **a** Complementarity determining regions (CDRs) and predicted antigen contact structure (ACS) for protective (mAb8) and detrimental (mAb9) anti-TN mAbs. Note the “cave-like” shape of mAb8’s ACS and the “groove-like” conformation of mAb9’s ACS. **b** Epitope mapping of mAb8 and mAb9 against a panel of TN peptides (P1 – P10) via dot blot analysis. While mAb8 binds P5, mAb9 exclusively targets P2, which correlates with their previously reported divergent effects on septic survival. **c** Predicted conformation and spatial arrangement of the P2 and P5 epitopes on the human TN. The ribbon (top) and space-fill models (bottom) illustrate that P2 and P5 are distinct, accessible epitopes on the TN protein surface, which is consistent with the predicted ACS. **d** Amino acid sequence of the P2 epitope and its P2-1 derivative. P2-1 was engineered to increase solubility by removing a water-insoluble phenylalanine (F) at the C-terminal residue 70, introducing a water-soluble threonine (T) at the N-terminal residue 54, and reducing oxidative susceptibility via the substitution of methionine (M) with norleucine (Nle) and cysteine (C) with serine (S). TN tetranectin, mAb monoclonal antibody

We previously reported that certain anti-TN mAbs, such as mAb8, protected mice against sepsis, while others, exemplified by mAb9, paradoxically exacerbated lethality [21], suggesting the existence of distinct functional epitopes. To explore the therapeutic potential of this detrimental epitope, we determined the amino acid sequences of mAb8 and mAb9 and computationally predicted their ACS (Fig. 1a). The ACS of mAb8 displayed a “cave-like” shape, well-suited for a protruding antigen epitope. In contrast, mAb9’s ACS exhibited a “groove-like” conformation, implying binding to a linear epitope. Systematic dot-blotting using 10 synthesized TN peptides (P1 – P10, Additional file 1: Fig. S4a, b) revealed that mAb8 targeted P5, while mAb9 exclusively interacted with P2 (Fig. 1b). Structural modeling further confirmed P2 and P5 as spatially separated (Fig. 1c, top panel) and accessible (Fig. 1c, bottom panel) epitopes on the TN protein surface, consistent with the predicted ACS (Fig. 1a). This structural validation revealed that targeting different TN epitopes leads to divergent functional outcomes. To enhance therapeutic applicability, we engineered P2-1 to: 1) improve solubility by removing a water-insoluble phenylalanine (F) at C-terminal residue 70 and introducing a water-soluble threonine (T) at N-terminal residue 54; and 2) reduce oxidative susceptibility by substituting methionine (M) with norleucine (Nle) and cysteine (C) with serine (S), respectively (Fig. 1d).

### The C-terminal CRD of TN retained protective properties in sepsis

Given TN’s diverse functional domains (Additional file 1: Fig. S4a) and our P2 epitope findings, we sought to delineate its core protective mechanism. We generated a recombinant TN mutant (ΔTN, residues 45–181) lacking the N-terminal heparin binding and α-helix trimerization regions (Additional file 1: Fig. S4c), and evaluated its therapeutic potential in the CLP model of sepsis. As shown in Additional file 1: Fig. S4d, ΔTN conferred significant protection against lethal sepsis, comparable to full-length TN [21]. This indicates that TN’s protective functionality was largely retained within its C-terminal CRD, which notably encompasses the P2 epitope (residues 55–70, Additional file 1: Fig. S4a, b). This finding suggested that the oligomeric state, mediated by the N-terminal trimerization region, might not be essential for TN’s protective effects. To pinpoint the minimal active region for these benefits, we explored the protective capacities of 10 synthetic peptides from the human TN CRD (P1 – P10, Additional file 1: Fig. S4b), including P2, in the CLP sepsis model.

### P2 and P2-1 derivatives conferred significant protection against lethal sepsis

**Fig. 2:**
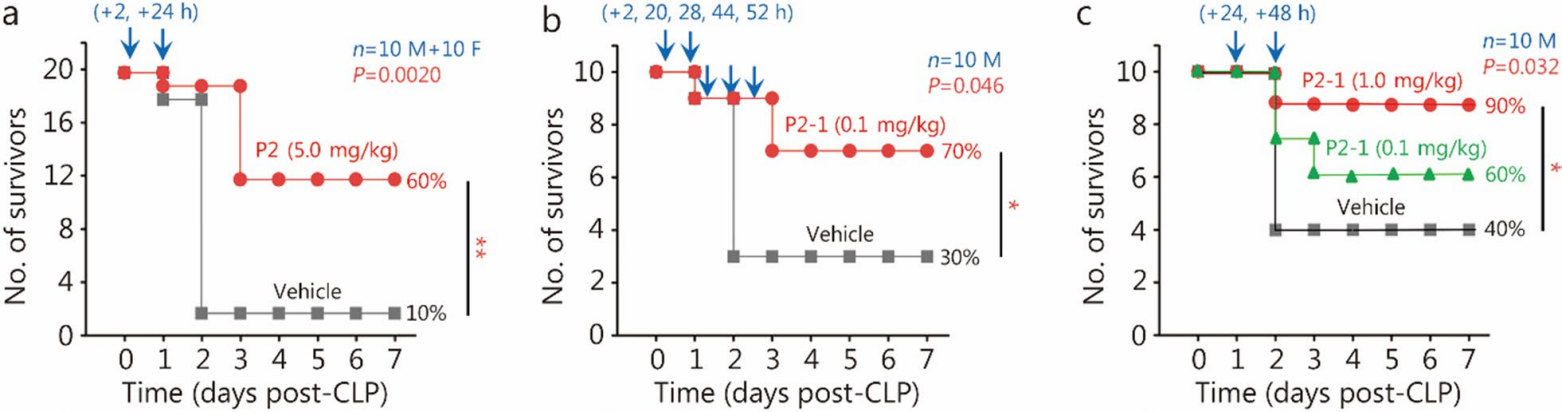
P2 and its P2-1 derivative conferred significant protection against lethal sepsis. **a** Repetitive administration of the P2 peptide [5.0 mg/kg at 2 h and 24 h post-cecal ligation and puncture (CLP)] significantly increased animal survival in male (“M”) and female (“F”) mice. **b** P2-1 derivative (0.1 mg/kg, administered at 2, 20, 28, 44, and 52 h post-CLP) also conferred significant protection against lethal sepsis. **c** Delayed administration of P2-1 (1.0 mg/kg at 24 h and 48 h post-CLP) rescued mice from lethal sepsis. ^*^P < 0.05, ^**^P < 0.01

The identification of the P2 epitope as the binding site for mAb9, which paradoxically reduced septic survival [21], presents a compelling therapeutic opportunity. We evaluated the therapeutic efficacy of both P2 and its water-soluble derivative, P2-1, in CLP-induced sepsis. Repetitive administration of P2 (5.0 mg/kg twice at 2 h and 24 h post-CLP) significantly increased survival rates in both male and female mice (Fig. 2a). Similarly, P2-1, at a lower dose (0.1 mg/kg) and at multiple time points (e.g., 2, 20, 28, 44, and 52 h post-CLP), also conferred significant protection against sepsis (Fig. 2b), indicating that P2-1 retained P2’s efficacy with improved pharmacological properties. Crucially, P2-1 provided potent protection against lethal sepsis even with delayed therapeutic administration (24 h and 48 h post-CLP) at a dose (1.0 mg/kg) approximately fivefold lower than that of P2 (Fig. 2c). The ability of P2-1 to protect a delayed therapeutic setting is particularly significant for clinical sepsis, where early intervention is often challenging.

**Fig. 3:**
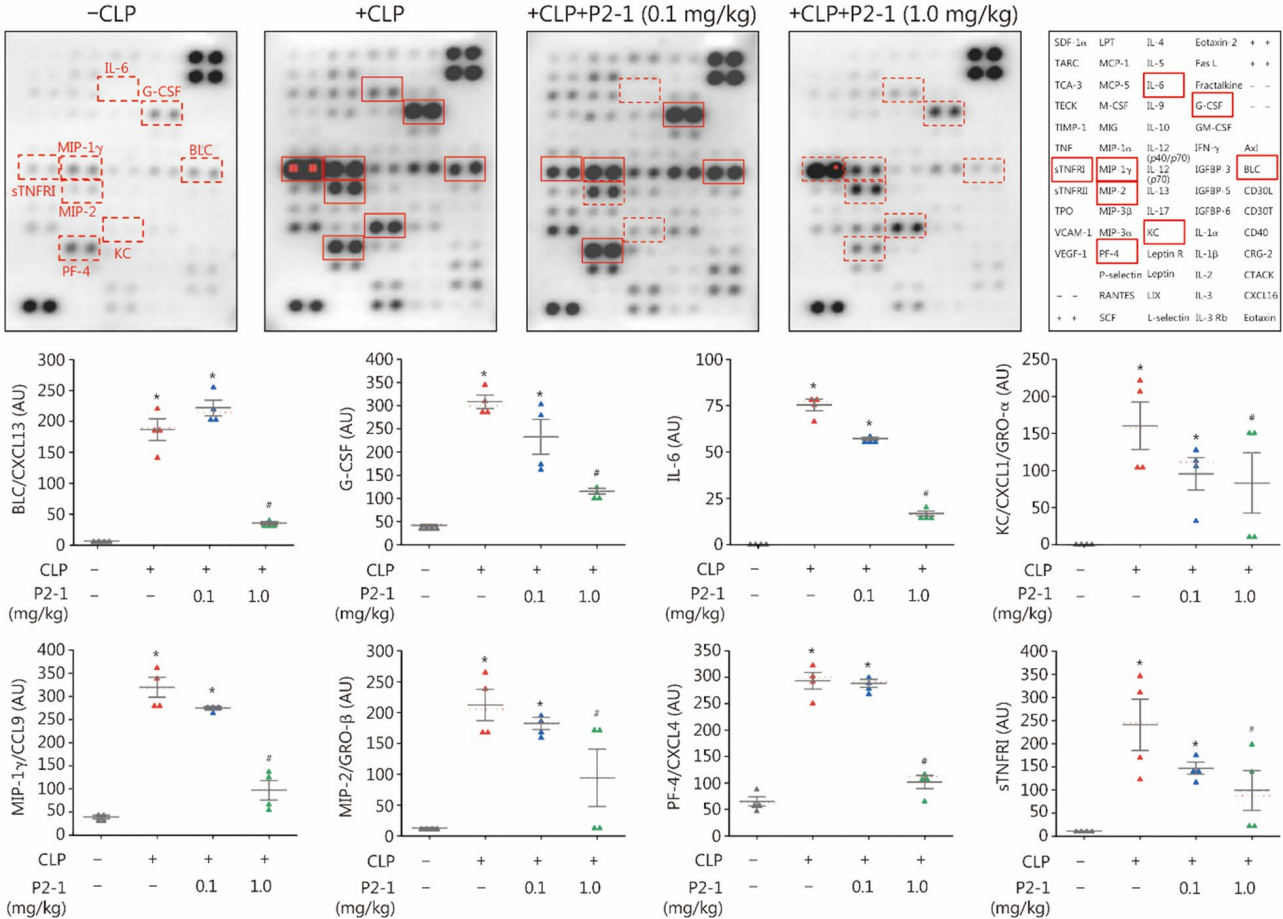
P2-1 significantly attenuated sepsis-induced systemic inflammation. BALB/c mice subjected to CLP-induced sepsis received P2-1 (0.1 or 1.0 mg/kg) intraperitoneally twice, at 2 h and 20 h post-CLP. At 24 h post-CLP, the animals were sacrificed, and blood was harvested for cytokine and chemokine measurements via cytokine antibody arrays. ^*^P < 0.05 vs. the negative control (“- CLP”); ^#^*P* < 0.05 vs. the positive control (“ + CLP”) group, non-parametric Kruskal-Wallis ANOVA test. CLP cecal ligation and puncture, BLC B-lymphocyte chemoattractant, CCL C–C motif chemokine ligand, CXCL C-X-C motif chemokine ligand, IL interleukin, G-CSF granulocyte colony-stimulating factor, KC keratinocyte-derived chemokine, GRO growth-regulated oncogene, MIP macrophage inflammatory protein, PF platelet factor, sTNFR soluble tumor necrosis factor receptor

To investigate P2-1’s protective mechanisms, we assessed its effects on sepsis-induced systemic inflammation. Intraperitoneal P2-1 administration significantly reduced sepsis-induced systemic accumulation of B-lymphocyte chemoattractant (BLC)/chemokine (C-X-C motif) ligand (CXCL) 13, granulocyte colony-stimulating factor (G-CSF), IL-6, keratinocyte-derived chemokine (KC)/CXCL1/growth-regulated oncogene (GRO)-α, macrophage inflammatory protein-1γ (MIP-1γ/chemokine (C–C motif) ligand (CCL) 9, MIP-2/GRO-β, platelet factor 4 (PF-4)/CXCL4, and soluble tumor necrosis factor receptor I (sTNFRI) (Fig. 3). This comprehensive reduction in a broad panel of proinflammatory cytokines and chemokines resembled the effect of pCTS-L-neutralizing antibodies in sepsis [5]. It suggests that P2-1 confers protection against sepsis by potentially mitigating pCTS-L-mediated systemic inflammation and the accumulation of key surrogate biomarkers, observed in experimental sepsis [57, 58].

### P2-1 conferred a dose-dependent protection against CAIA

**Fig. 4:**
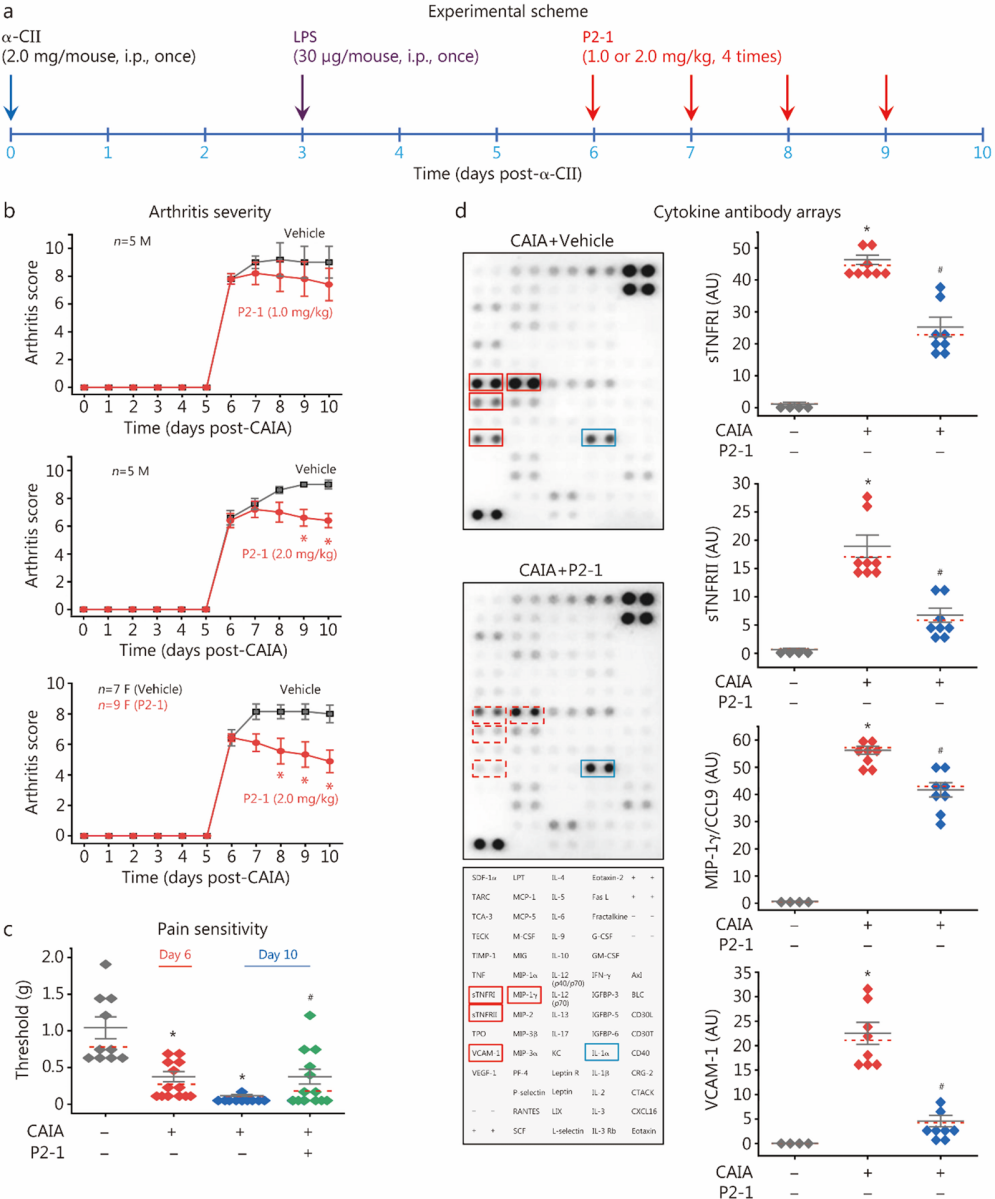
P2-1 conferred a dose-dependent protection against collagen antibody-induced arthritis (CAIA). **a** Experimental scheme for CAIA induction and P2-1 therapeutic intervention in BALB/c mice. **b** P2-1 significantly attenuated CAIA-induced arthritis severity in both male (“M”) and female (“F”) mice. ^*^P < 0.05 vs. the vehicle group, non-parametric Kruskal-Wallis ANOVA test. **c** P2-1 significantly reduced CAIA-induced pain sensitivity by partially restoring paw mechanical withdrawal thresholds. ^*^P < 0.05 vs. the nonarthritic negative control (“- CAIA”); ^#^*P* < 0.05 vs. vehicle-treated CAIA control (“ + CAIA”) on the same day. **d** P2-1 reduced CAIA-induced joint tissue inflammation. Cytokine antibody arrays of joint tissue lysates (harvested on Day 10) revealed reduced levels of sTNFRI, sTNFRII, MIP-1γ, and VCAM-1 in P2-1-treated mice. IL-1α served as a negative control. ^*^P < 0.05 vs. nonarthritic negative control (“- CAIA”); ^#^*P* < 0.05 vs. vehicle-treated CAIA control (“ + CAIA”), non-parametric Kruskal-Wallis ANOVA test. LPS lipopolysaccharides, STNFRI soluble tumor necrosis factor receptor, VCAM vascular cell adhesion molecule, MIP macrophage inflammatory protein

Extending our success in experimental sepsis, we further evaluated P2-1’s efficacy in a CAIA model. Although the CAIA model may not fully capture the multifaceted process of joint bone destruction in human RA, it is an excellent model of the downstream inflammatory phase. This makes it ideal for studying the innate immune-driven synovitis, pain, and inflammatory tissue damage mediated by macrophages and neutrophils. Furthermore, microbial infections can often amplify inflammatory responses in joints, driving the onset and progression of RA [59]. As depicted in Fig. 4a, CAIA was induced with α-CII on Day 0, followed by LPS on Day 3 to activate inflammatory responses and synchronize disease onset. As a therapeutic intervention for established disease, P2-1 administration began on Day 6 post-α-CII challenge and continued daily for 4 consecutive days. Notably, P2-1 dose-dependently and significantly attenuated CAIA-induced arthritis severity in both male (“M”) and female (“F”) mice (Fig. 4b), highlighting its broad applicability across sexes.

Since RA is also characterized by significant pain responses, which are major drivers of patient morbidity and reduced quality of life, we assessed mechanical allodynia (pain sensitivity) via Von Frey filaments. As shown in Fig. 4c, P2-1 significantly reduced CAIA-induced pain sensitivity by partially restoring withdrawal thresholds to mechanical stimuli. Furthermore, P2-1 significantly reduced CAIA-induced joint tissue inflammation (Fig. 4d; Additional file 1: Fig. S5), as evidenced by the significant reduction in sTNFRI and sTNFRII, the chemokine MIP-1γ/CCL9, and vascular cell adhesion molecule-1 (VCAM-1) in the joint tissue. These findings collectively suggest that P2-1 exerts its protective effects by dampening the inflammatory milieu within the affected joints, thereby ameliorating both clinical disease progression and pain.

When administered prophylactically before CAIA induction (Additional file 1: Fig. S6a), P2-1 significantly reduced arthritis severity, with initial improvements noted as early as Day 6 and a significant reduction observed by Day 7 post-α-CII challenge (Additional file 1: Fig. S6b). This prophylactic strategy also significantly reduced CAIA-induced pain sensitivity through partial restoration of mechanical withdrawal thresholds (Additional file 1: Fig. S6c). Similarly, prophylactic P2-1 administration attenuated joint inflammation, significantly reducing joint levels of sTNFRI and sTNFRII, the chemokine MIP-1γ/CCL9, and VCAM-1 (Additional file 1: Fig. S6d). Collectively, these findings demonstrate that P2-1 offers not only therapeutic potential but also a robust preventive effect against CAIA.

### P2-1 interacted with HMGB1 to selectively block HMGB1-induced *Ctsl* mRNA upregulation and pCTS-L secretion

**Fig. 5:**
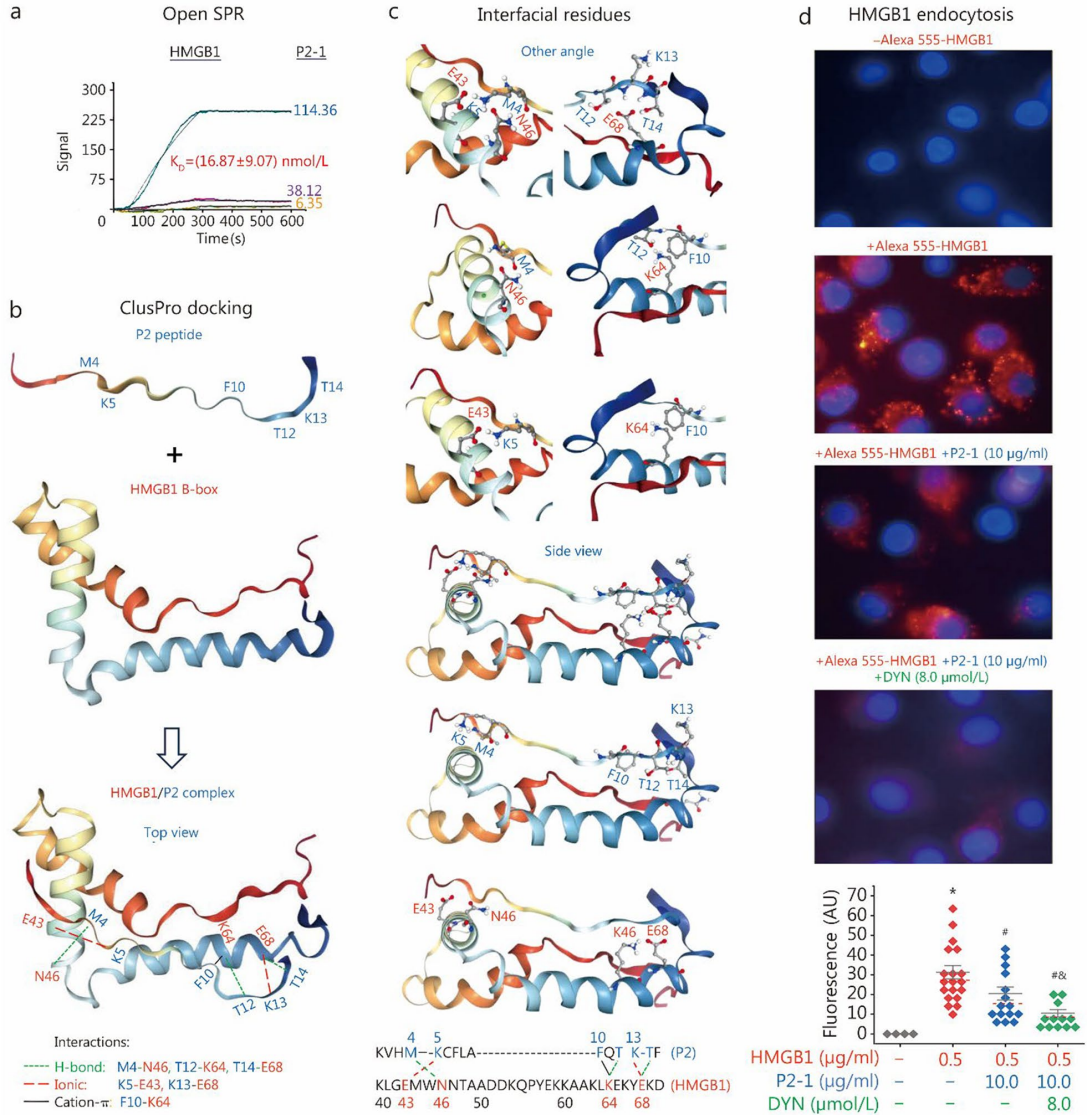
P2-1 interacted with HMGB1 and inhibited its cellular uptake. **a** SPR analysis of the P2-1/HMGB1 interaction, revealing dose-dependent binding of P2-1 to immobilized HMGB1 with an estimated K_D_ of (16.87 ± 9.07) nmol/L (mean ± SEM, *n* = 3 independent experiments).** b** ClusPro protein–protein docking of the HMGB1/P2 complex, highlighting key hydrogen bonds, ionic, and cation-π interactions at the binding interface.** c** Detailed views of the interfacial residues in the HMGB1-P2 complex from various angles, illustrating the specific amino acid interactions. **d** P2-1 inhibited HMGB1 cellular uptake by macrophages. Differentiated human macrophages were treated with Alexa Fluor 555-labeled HMGB1 (0.5 µg/ml) in combination with P2-1 (10 µg/ml) or dynasore (DYN, 8.0 µmol/L) for 2 h. Following washing and fixation, HMGB1 uptake was quantified as the mean fluorescence intensity of Alexa Fluor 555 in representative cells via ImageJ software. ^*^P < 0.05 vs. the negative control (“- Alexa 555-HMGB1”); ^#^*P* < 0.05 vs. the positive control (“ + Alexa 555-HMGB1”); ^&^*P* < 0.05 vs. the “ + Alexa 555-HMGB1 + P2-1” group, non-parametric Kruskal-Wallis ANOVA test. SPR surface plasmon resonance, HMGB1 high mobility group box 1, K_D_ dissociation equilibrium constant, SEM standard error of the mean

Prompted by P2-1’s consistent therapeutic efficacy in both sepsis and CAIA, we investigated its underlying molecular mechanism. We hypothesized that P2-1, derived from its precursor TN, exerts beneficial effects by directly binding pathogenic HMGB1 to modulate its detrimental functions. SPR confirmed P2-1’s high-affinity binding to HMGB1, with an equilibrium dissociation constant (K_D_) of (16.87 ± 9.07) nmol/L (Fig. 5a; Additional file 1: Fig. S3). To gain structural insights, we predicted the P2 peptide structure (Additional file 1: Fig. S3a; Additional file 2) and performed protein–protein docking with the human HMGB1 B-box domain (residues 89–167). This docking model (Fig. 5b; Additional file 1: Fig. S3b and Additional file 3) revealed that P2 directly interacted with the HMGB1 B-box (Additional file 1: Fig. S3c), a region critical for HMGB1’s proinflammatory functions [14]. Notably, key residues involved in P2 interactions (e.g., E43, N46, K64, and E68 of the HMGB1 B-box, Fig. 5b, c; or E131, N134, K152, and E156 of HMGB1, Additional file 1: Fig. S3c) were spatially distant from its Toll-like receptor 4 (TLR4)-binding domain (residues 89–108, Additional file 1: Fig. S3c), but partially overlapped with the RAGE-binding region (residues 150–183, Additional file 1: Fig. S3c) [60, 61]. This spatial relationship suggests that P2-1, by binding to this P2-interacting region of HMGB1, can selectively influence RAGE-dependent HMGB1 activities (e.g., HMGB1 endocytosis and macrophage pyroptosis) without impacting TLR4-dependent functions (e.g., induction of cytokines and chemokines).

To investigate P2-1’s effect on HMGB1 endocytosis, differentiated human macrophages were exposed to Alexa Fluor 555-labeled HMGB1 under various conditions (Fig. 5d). As previously reported [21], HMGB1 exposure resulted in substantial cellular uptake, characterized by prominent intracellular red fluorescence puncta and a significant increase in the mean fluorescence intensity in these cells (Fig. 5d). In sharp contrast to full-length TN, which significantly increased HMGB1 uptake [21], coincubation with P2-1 (10 µg/ml) markedly reduced Alexa Fluor 555-HMGB1 uptake, as confirmed by a significant decrease in the mean fluorescence intensity (Fig. 5d). Furthermore, combining P2-1 (10 µg/ml) with the endocytosis inhibitor dynasore (DYN, 8.0 µmol/L) led to an even greater reduction in HMGB1 uptake (Fig. 5d), suggesting that HMGB1 internalization by macrophages is likely mediated by endocytosis [17, 21]. Collectively, these findings demonstrate that P2-1 specifically binds HMGB1 to competitively inhibit the RAGE-HMGB1 interaction and RAGE-dependent HMGB1 endocytosis.

**Fig. 6:**
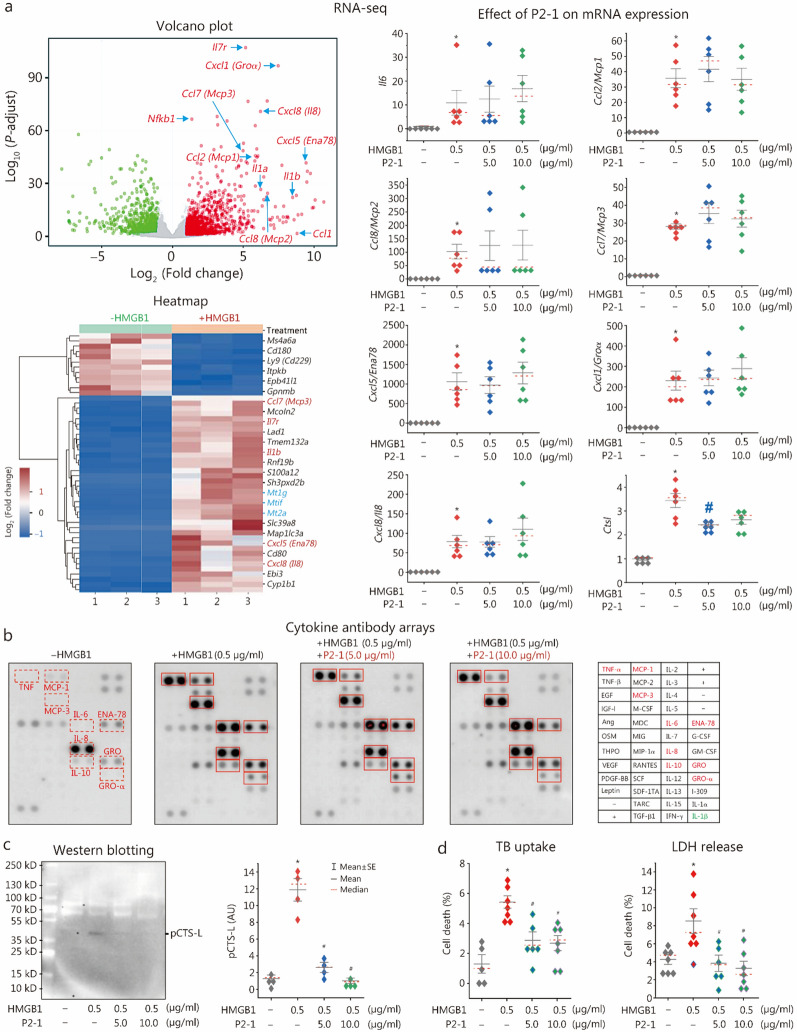
P2-1 selectively inhibited HMGB1-induced *Ctsl* expression and pCTS-L secretion in human immune cells. **a** RNA-seq analysis of gene expression in human PBMCs from three independent donors, stimulated with HMGB1 in the absence or presence of P2-1. Quantitative analysis of mRNA expression demonstrated that P2-1 significantly reduced HMGB1-induced *Ctsl* upregulation, but did not significantly affect the expression of other HMGB1-induced cytokines and chemokines. **b** Cytokine antibody array analysis of HMGB1-induced cytokine and chemokine secretion in human PBMCs. P2-1 (5.0 or 10.0 µg/ml) did not affect the secretion of these HMGB1-induced cytokines or chemokines. **c** Western blotting analysis of HMGB1-induced pCTS-L secretion in human PBMCs. P2-1 dose-dependently abrogated HMGB1-induced pCTS-L secretion (*n* = 4 independent experiments). ^*^P < 0.05 vs. the negative control (“- HMGB1”); ^#^*P* < 0.05 vs. the positive control (“ + HMGB1”), non-parametric Kruskal-Wallis ANOVA test. **d** Trypan blue (TB) uptake and LDH release assays of HMGB1-induced macrophage death. P2-1 significantly inhibited HMGB1-induced cell death in differentiated human macrophages, as evidenced by reduced TB uptake and LDH release. ^*^P < 0.05 vs. the negative control (“- HMGB1”); ^#^*P* < 0.05 vs. the positive control (“ + HMGB1”), non-parametric Kruskal-Wallis ANOVA test. HMGB1 high mobility group box 1, Ctsl cathepsin L, pCTS-L procathepsin L, RNA-Seq RNA sequencing, PBMCs peripheral blood mononuclear cells, LDH lactate dehydrogenase, CCL chemokine (C–C motif) ligand, MCP monocyte chemoattractant protein, CXCL chemokine (C-X-C motif) ligand, GRO growth-regulated oncogene

To identify P2-1’s downstream targets, we performed RNA-seq analysis on human PBMCs stimulated with HMGB1, with or without P2-1 cotreatment. The volcano plot and heatmap (Fig. 6a) revealed that HMGB1 stimulation profoundly upregulated many inflammatory cytokines (e.g.,* Il1b*) and chemokines (e.g., *Cxcl5/Ena78*, *Ccl7/Mcp3*, and *Cxcl8/Il8*) (Additional file 4), validating HMGB1’s critical role as an inflammatory mediator of sepsis and RA [6, 61, 62]. However, P2-1 did not broadly reverse HMGB1-induced transcriptional changes in most genes (Additional file 1: Fig. S7a, b), including highly upregulated inflammatory cytokines (e.g., *Il6*) and chemokines (e.g., *Ccl2/Mcp1, Ccl8/Mcp2, Ccl7/Mcp3, Cxcl5/Ena78, Cxcl1/Groα, Cxcl8/Il8*) (Fig. 6a, right panels; Additional file 4). In sharp contrast, P2-1 treatment resulted in a moderate yet significant reduction in HMGB1-induced *Ctsl* mRNA upregulation, with levels decreasing from (3.4 ± 0.3)-fold (in the HMGB1-only group) to (2.4 ± 0.1)-fold (in the HMGB1 + P2-1 group, 5.0 µg/ml) (Fig. 6a, right panel; Additional file 4).

To validate these RNA-seq findings, we quantified HMGB1-induced cytokine/chemokine protein levels using cytokine antibody arrays. Consistent with our RNA-seq data, HMGB1 stimulation led to a significant increase in the protein levels of various cytokines (e.g., TNF, IL-6, and IL-10) and chemokines (e.g., CXCL5/ENA-78, CXCL1/GRO-α, CXCL8/IL-8, CCL2/MCP-1, and CCL7/MCP-3) in human PBMCs (Fig. 6b), confirming its potent immunomodulating activities [62]. However, P2-1 did not broadly inhibit the secretion of these HMGB1-induced cytokines and chemokines (Fig. 6b; Additional file 1: Fig. S8), underscoring the selectivity of its action. This selectivity aligns with previous findings that TN, P2-1’s precursor, also did not affect HMGB1-induced cytokines (e.g., IL-6) or chemokines (e.g., MCP-1) [21]. In stark contrast, Western blotting analysis revealed a striking and dose-dependent suppression of HMGB1-induced pCTS-L secretion by P2-1 in human PBMCs (Fig. 6c; Additional file 1: Fig. S9). These findings, coupled with our RNA-seq results, collectively demonstrate that P2-1 selectively inhibits HMGB1-induced pCTS-L secretion, highlighting a pathogenic role for the HMGB1-pCTS-L axis in sepsis and RA.

Although P2-1 did not affect HMGB1-induced cytokine or chemokine production (Fig. 6b; Additional file 1: Fig. S8), its specific inhibition of the HMGB1-RAGE interaction (Additional file 1: Fig. S3c) and subsequent RAGE-dependent HMGB1 endocytosis (Fig. 5d) strongly suggested its potential to suppress HMGB1-induced macrophage pyroptosis. Indeed, P2-1 significantly inhibited HMGB1-induced macrophage death, as evidenced by reduced trypan blue uptake and LDH release (Fig. 6d). This observed preservation of cell membrane integrity suggests that P2-1 directly interferes with HMGB1-RAGE-dependent macrophage pyroptosis.

### A pCTS-L-neutralizing mAb protected against CAIA

**Fig. 7:**
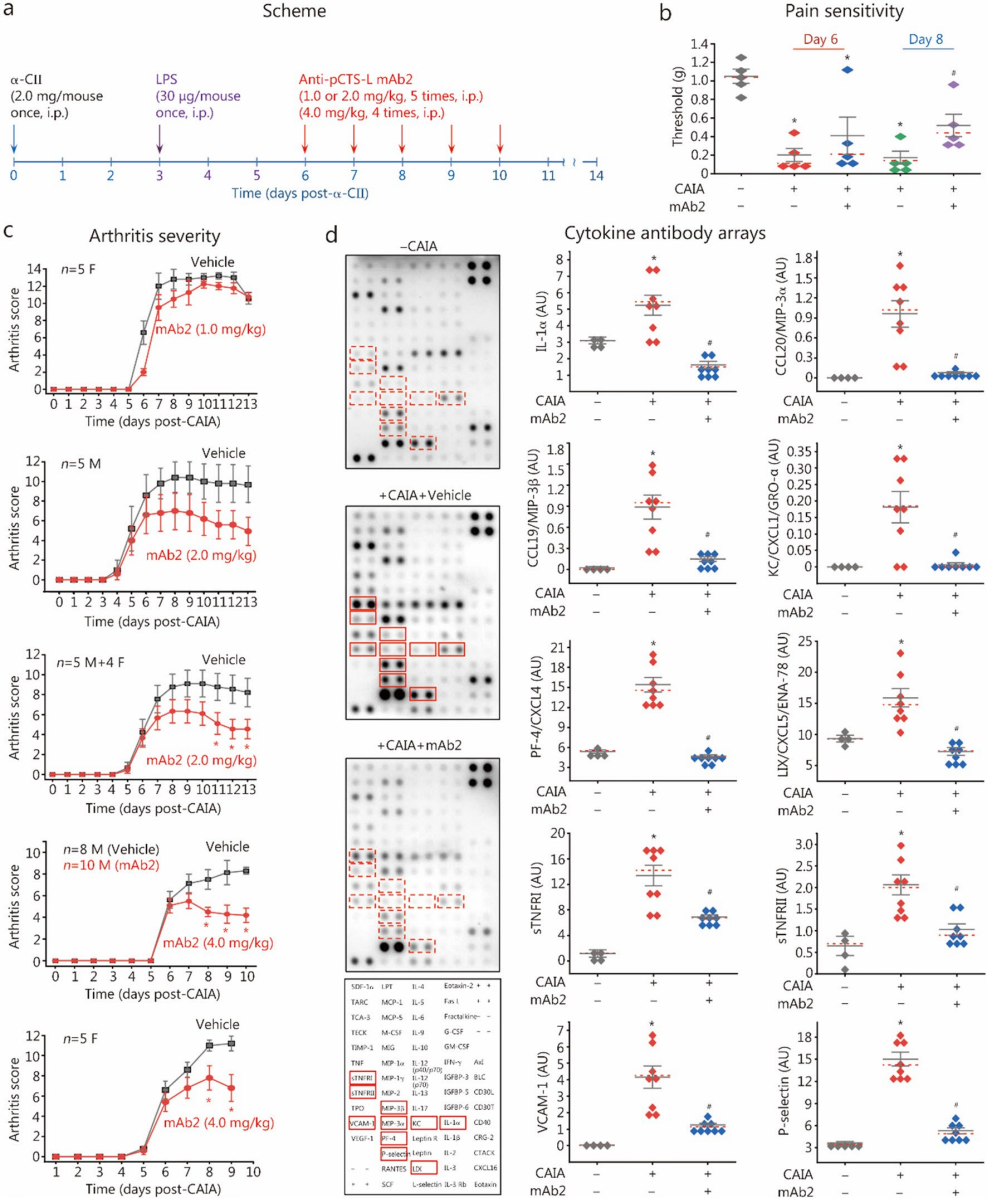
A pCTS-L-neutralizing mAb (mAb2) conferred dose-dependent protection against collagen antibody-induced arthritis (CAIA). **a** Experimental scheme for CAIA induction and pCTS-L-neutralizing mAb2 therapeutic intervention in BALB/c mice. **b** mAb2 (2.0 mg/kg) significantly reduced CAIA-induced pain sensitivity by partially restoring paw mechanical withdrawal thresholds. ^*^P < 0.05 vs. the nonarthritic negative control (“- CAIA”); ^#^*P* < 0.05 vs. the vehicle-treated CAIA control (“ + CAIA”) on the same day, non-parametric Kruskal-Wallis ANOVA test. **c** mAb2 significantly attenuated CAIA-induced arthritis severity in both male (“M”) and female (“F”) mice. ^*^P < 0.05 vs. vehicle control group, non-parametric Kruskal-Wallis ANOVA test. **d** mAb2 (2.0 mg/kg) reduced CAIA-induced joint tissue inflammation. ^*^P < 0.05 vs. non-arthritic negative control (“- CAIA”); ^#^*P* < 0.05 vs. the vehicle-treated CAIA control (“ + CAIA”), non-parametric Kruskal-Wallis ANOVA test. LPS lipopolysaccharides, pCTS-L procathepsin L, STNFRI soluble tumor necrosis factor receptor 1, CCL chemokine (C–C motif) ligand, CXCL chemokine (C-X-C motif) ligand, IL interleukin, KC keratinocyte-derived chemokine, LIX LPS-induced CXC chemokine, PF platelet factor, MIP macrophage inflammatory protein

Given pCTS-L’s pathogenic role in sepsis [5, 6, 24–26] and its elevation in experimental animals [30, 31, 63] and patients with RA [27, 29], we evaluated the therapeutic potential of a pCTS-L-neutralizing mAb in a CAIA model of RA (Fig. 7a). Administration of the pCTS-L-neutralizing mAb2 significantly mitigated CAIA-induced pain sensitivity (Fig. 7b), as evidenced by a partial normalization of withdrawal thresholds to mechanical stimulation. Consistently, mAb2 dose-dependently and significantly attenuated CAIA-induced arthritis severity in both male (“M”) and female (“F”) mice (Fig. 7c), mirroring the broad efficacy of P2-1, a selective pCTS-L inhibitor, in preclinical settings.

Direct extrapolation of therapeutic doses from mouse models to humans is highly complex because of significant species-specific differences in physiology, pharmacokinetics (PK), pharmacodynamics (PD), and safety profiles. To address this, formal PK/PD studies will be conducted in at least two animal species (e.g., mouse and non-human primate). This will allow us to build an allometric scaling model to more accurately predict the human equivalent dose, clearance rate, and the dosing frequency required to maintain therapeutic concentrations. Nevertheless, a simple weight-based scaling of our mouse dose (2.0 mg/kg for mAb2) to a 70 kg human yields an estimated 140 mg. This, however, might be an overestimation, because: 1) Fc-modified humanized mAbs typically exhibit longer half-lives in humans [64], potentially allowing less frequent dosing; and 2) our preclinical study in mice used i.p. administration, which generally yields lower bioavailability than the clinically relevant intravenous route for human therapy [65]. Therefore, while our preclinical studies demonstrated the efficacy of anti-pCTS-L mAb2 at 2.0 mg/kg in both experimental sepsis [5] and RA (Fig. 7b, c), accurate human dose estimation will require dedicated preclinical testing, sophisticated modeling, and rigorous future clinical trials.

These symptomatic and clinical benefits were correlated with significant reductions in a range of proinflammatory mediators within the joint microenvironment (Fig. 7d; Additional file 1: Fig. S10), these included cytokines (e.g., IL-1α), chemokines (e.g., CCL20/MIP-3α, CCL19/MIP-3β, KC/CXCL1/GRO-α, PF-4/CXCL4, and LIX/CXCL5/ENA-78), sTNFRI and sTNFRII, and adhesion molecules (e.g., VCAM-1 and P-selectin). Collectively, these results demonstrate that the pCTS-L-neutralizing mAb2 provides robust therapeutic efficacy in the CAIA model, successfully ameliorating both the clinical manifestations of RA and associated pain by suppressing the local inflammatory cascade within inflamed joints.

### Histopathological evidence: pCTS-L-neutralizing mAb protected against arthritis and joint inflammation

**Fig. 8:**
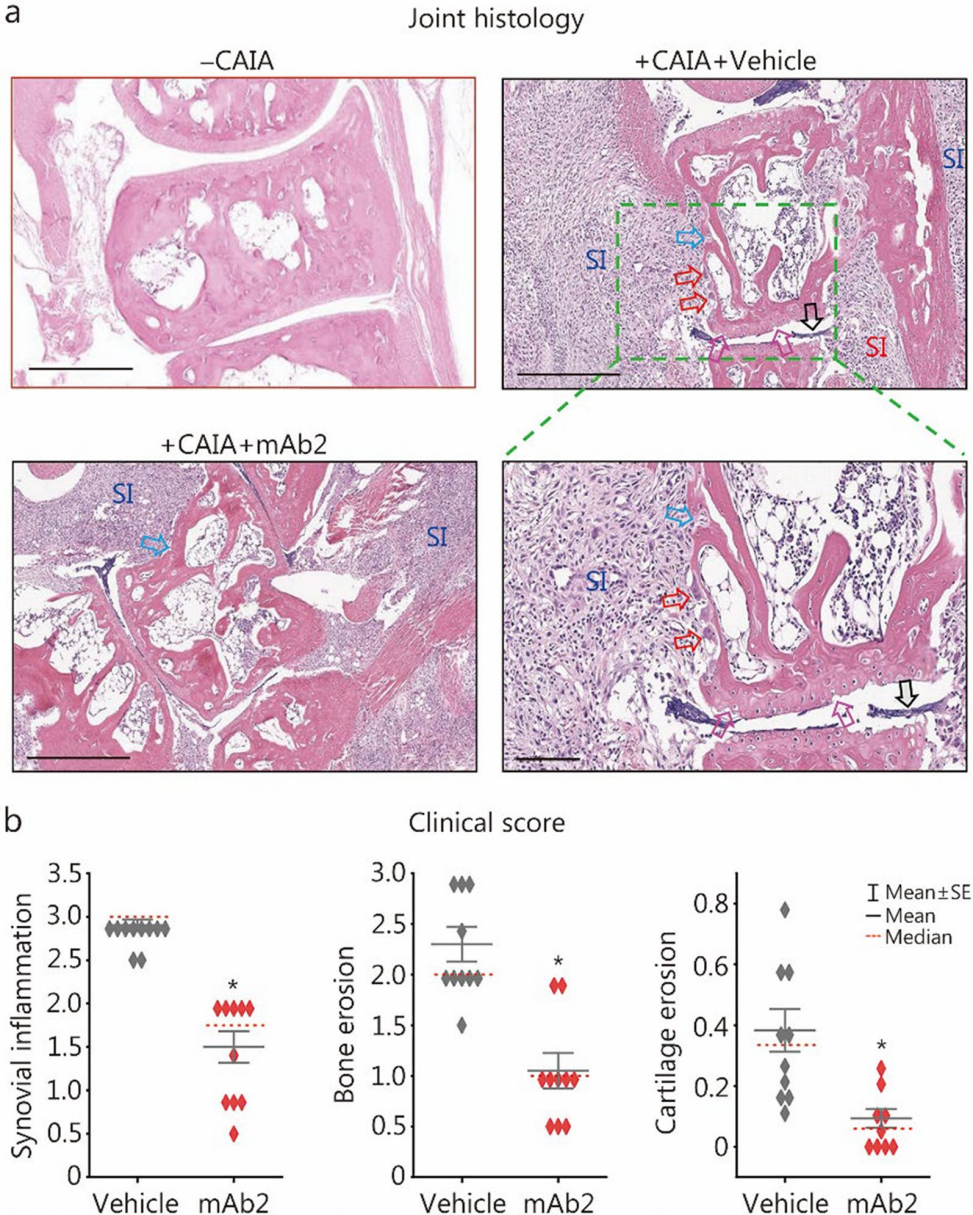
A pCTS-L-neutralizing mAb reduced ankle joint inflammation and structural damage in CAIA mice. **a** Representative histological analysis of H&E-stained ankle joints from normal (“- CAIA”), saline vehicle-treated CAIA (“ + CAIA + vehicle”), and pCTS-L-neutralizing mAb2-treated CAIA (“ + CAIA + mAb2”) mice. Vehicle-treated CAIA mice presented severe synovial joint inflammation (SI) characterized by extensive inflammatory cell infiltration, pronounced synoviocyte hyperplasia, thickening of synovial lining and sublining, and erosion of bone (blue arrow) and cartilage (purple arrows). The expanded synovial lining, combined with dense inflammatory infiltrates and activated fibroblasts in the sublining, collectively formed highly aggressive, tumor-like structures called “pannus” (black arrows). Pannus invasion of adjacent cartilage and bone resulted in osteoclast clustering on the bone surface (red arrows) and subsequent bone erosion. Note that mAb2 treatment profoundly attenuated these pathologies, preserving overall joint architecture. **b** Quantitative histological assessment of synovial inflammation, bone erosion, and cartilage erosion in mAb2-treated CAIA mice compared with vehicle control mice. ^*^P < 0.05 vs. the vehicle group, non-parametric Kruskal-Wallis ANOVA test. CAIA collagen antibody-induced arthritis, mAb2 monoclonal antibody 2, SE standard error

Consistent with these preclinical findings, histological analysis of ankle joints from vehicle-treated CAIA animals revealed profound synovial joint inflammation, marked by extensive inflammatory cell infiltration, pronounced synoviocyte hyperplasia, and substantial thickening of the synovial lining and sublining (Fig. 8a). The expanded synovial lining, combined with dense inflammatory infiltrates and activated fibroblasts in the sublining, collectively formed highly aggressive, tumor-like structures known as “pannus” (Fig. 8a, black arrow). This pannus could relentlessly invade and degrade adjacent cartilage and bone, promoting osteoclast clustering on the bone surface (red arrows) and subsequent erosion of bone (blue arrow) and cartilage (purple arrows). In striking contrast, pCTS-L-neutralizing mAb2 treatment profoundly attenuated these severe histological pathologies. Joints from mAb2-treated animals presented substantially reduced inflammatory cell infiltration, diminished synovial hyperplasia and thickening, and critically, amelioration of both bone and cartilage erosion (Fig. 8a). Compared with those of vehicle controls, quantitative histological scoring of synovial inflammation, bone erosion, and cartilage erosion further confirmed statistically significant reductions in all measured parameters in response to mAb2 treatment (Fig. 8b). The mAb2’s ability to attenuate both inflammation and structural damage suggests pCTS-L as a promising therapeutic target for disease-modifying interventions in human RA.

## Discussion

Sepsis and RA are distinct yet mechanistically related inflammatory conditions commonly driven by dysregulated innate immune responses and excessive cytokine production. Despite extensive research, effective sepsis therapies remain elusive, and existing RA treatments often suffer from limited efficacy and significant side effects. Our study introduces a paradigm-shifting drug discovery strategy: repurposing an epitope derived from a detrimental anti-TN antibody [21] into P2-1, a targeted therapy for both sepsis and RA. This counterintuitive approach originated from our observation that different anti-TN antibodies, which target different epitopes, yielded divergent functional outcomes [21]. We hypothesized that the P2 epitope, when administered as a standalone peptide, would serve as a decoy by directly competing with TN or RAGE for HMGB1 binding, thereby disrupting HMGB1’s detrimental functions (Additional file 1: Fig. S11).

Specifically, our study demonstrated that P2-1 directly binds HMGB1, a critical mediator in both sepsis [4, 18, 66, 67] and RA [9], through specific interactions with key residues (E131, N134, K152, and E156) within HMGB1’s B-box domain. Notably, these P2-binding sites are spatially separated from HMGB1’s TLR4-binding region (residues 89–108) but partially overlap with the RAGE-binding domain (residues 150–183). Based on this receptor-specific spatial geometry, we propose a model to explain P2-1’s precision targeting of the HMGB1-RAGE-pCTS-L axis. Specifically, P2-1 does not disrupt HMGB1-TLR4 binding, thereby preserving the broad TLR4-dependent induction of cytokines and chemokines [14, 15]. In contrast, P2-1 is hypothesized to selectively disrupt HMGB1-RAGE signaling, a pathway implicated in HMGB1 endocytosis, macrophage pyroptosis [17, 18], and potentially pCTS-L induction. This proposed RAGE-specific interference would account for P2-1’s precise inhibition of HMGB1-induced pCTS-L induction without broadly affecting other cytokines and chemokines. Nevertheless, future investigations are needed to definitively determine whether P2-1’s selective suppression of pCTS-L is abrogated by pharmacological or genetic blockade of RAGE or other off-target receptors (Additional file 1: Fig. S11). Such studies will establish the receptor-specific mechanistic basis for P2-1’s therapeutic precision.

Indeed, P2-1 did not broadly suppress HMGB1-induced cytokines and chemokines, but selectively inhibited HMGB1-mediated *Ctsl* mRNA upregulation and pCTS-L secretion, thus functioning as a precise inhibitor of the HMGB1-pCTS-L axis (Additional file 1: Fig. S11). Furthermore, P2-1 effectively blocked HMGB1 endocytosis and subsequent macrophage death, thereby specifically preventing its detrimental functions, which include pCTS-L secretion and innate immune cell depletion (Additional file 1: Figs. S1, S11). This approach not only offers a sophisticated strategy for modulating pervasive inflammatory pathways but also underscores the value of re-evaluating initial negative biological observations for uncovering hidden therapeutic potential in drug discovery.

Crucially, our findings reveal a striking dichotomy in the biological effects of full-length TN and its derived peptide, P2-1, on HMGB1-mediated detrimental macrophage responses. Previously, we demonstrated that TN enhances HMGB1 endocytosis and subsequent macrophage pyroptosis [21]. In contrast, our current data showed that P2-1 inversely inhibited HMGB1 uptake and HMGB1-induced macrophage pyroptosis. We propose that full-length TN, when complexed with HMGB1, may engage unidentified macrophage surface receptor(s), thereby facilitating receptor-mediated endocytosis of TN/HMGB1 complexes and activating pro-pyroptotic signaling. Conversely, P2-1, a smaller peptide derived from TN, retains the HMGB1-binding motif but lacks the essential domains required for engaging these pro-endocytic macrophage receptors. Therefore, while TN acts as an “on-switch” for TN/HMGB1 complex internalization, P2-1 functions as an “off-switch” by binding HMGB1 to prevent its interaction with pro-endocytic receptors (e.g., RAGE), thereby suppressing HMGB1 uptake and macrophage pyroptosis (Additional file 1: Figs. S1, S11). This distinct mechanism positions P2-1 as a more promising therapy to specifically block HMGB1-mediated detrimental events, including pCTS-L upregulation and macrophage pyroptosis.

Moreover, P2-1 achieves therapeutic specificity by targeting HMGB1 precisely where it is pathogenic. In healthy tissues, HMGB1 remains sequestered within the nucleus [6], rendering P2-1 pharmacologically inert within these healthy microenvironments. Conversely, upon inflammation, cellular stress induces HMGB1’s extracellular release [6], which becomes a hallmark of a pathological microenvironment and provides a pathology-specific target for P2-1. This “pathology-activated” mechanism, therefore, spatially restricts P2-1’s immunomodulatory activity to disease sites, offering a superior safety advantage over other systemic immunosuppressants.

Furthermore, the significance of this selective mechanism was further underscored by our direct validation of pCTS-L as a therapeutic target. While pCTS-L’s role as a late-acting mediator of lethal sepsis was recently identified [5, 6], its direct involvement and therapeutic potential in RA have been less explored. Here, we established a mechanistic link between HMGB1 and pCTS-L by demonstrating that HMGB1, a critical mediator of sepsis [4, 18, 66, 67] and RA [9, 10], induced pCTS-L expression and secretion in human immune cells (Additional file 1: Fig. S11). Furthermore, a pCTS-L-neutralizing antibody (mAb2) significantly attenuated arthritis and pain in the CAIA model, profoundly ameliorating joint inflammation, cartilage destruction, and bone erosion. The ability of a neutralizing antibody to mitigate RA progression positions pCTS-L as a highly promising and novel therapeutic target, potentially offering an alternative pathway beyond current anti-TNF therapies. Our findings align with previous observations of elevated CTS-L levels in animal models [30, 31, 63] and RA patients [27–29], and agree with the protective effects of genetic or pharmacological *Ctsl* suppression in experimental arthritis models [32, 33].

Importantly, a notable aspect of this research is the successful translation of a therapeutic strategy from acute sepsis to chronic RA, two distinct diseases that share common inflammatory pathways involving key mediators such as TNF, HMGB1, and pCTS-L (Additional file 1: Fig. S11). The consistent efficacy of P2-1 in both models, even with delayed therapeutic administration, underscores its potential as a selective anti-inflammatory agent for the HMGB1-pCTS-L axis. The ability of P2-1 to reduce systemic inflammatory mediators in sepsis (e.g., IL-6, KC/CXCL1/GRO-α, and MIP-2/GRO-β/CXCL3) and local joint inflammation in RA (e.g., sTNFRI, sTNFRII, MIP-1γ/CCL9, and VCAM-1) reinforces its immunomodulatory effects against key inflammatory mediators within the HMGB1-pCTS-L axis (Additional file 1: Fig. S11).

To that end, our initial focus on sepsis and RA was strategic, as these conditions serve as excellent models for acute systemic and chronic localized inflammation, respectively. The demonstrated efficacy in both models validates the central importance of the HMGB1-pCTS-L axis across diverse inflammatory conditions. This validation strongly suggests that P2-1’s therapeutic utility may extend well beyond sepsis and RA to other pathological conditions driven by similar immunopathological pathways. These could include acute inflammatory conditions such as ischemia-reperfusion injury and trauma, as well as chronic diseases like systemic lupus erythematosus, inflammatory bowel disease, and neuroinflammatory disorders. Therefore, this cross-disease study serves as a crucial proof-of-concept, establishing a compelling therapeutic rationale for evaluating agents that target the HMGB1-pCTS-L axis across a broad spectrum of inflammatory diseases.

In this context, our study design was intentionally calibrated to test two classes of disease-modifying agents (P2-1 and anti-pCTS-L mAb) in models of high but sub-maximal severity, reflecting clinically relevant scenarios where intervention remains viable. For sepsis, we employed a CLP model exhibiting 60–90% lethality, a severe challenge that avoids the pharmacological intractability of uniformly (100%) lethal models. For arthritis, the CAIA model was induced to an advanced disease (clinical score of approximately 8–10) that critically preceded irreversible joint destruction observed in maximal-severity models (clinical score of 16). The robust efficacy observed in these stringent models, improving survival in sepsis and attenuating disease progression in arthritis, provides a strong rationale for the clinical development of these agents for severe inflammatory diseases in the future.

Overall, our strategy is inspired by the successful translational path of anti-TNF therapies, which, though ineffective in clinical sepsis, became cornerstone treatments for RA by revealing fundamental pathogenic pathways [11, 12]. This historical precedent highlights that acute inflammation models can effectively illuminate fundamental pathways driving chronic autoimmune diseases [13]. Leveraging these cross-disease models, we have established P2-1 as a strong dual-indication candidate. For sepsis, P2-1 targets the late-acting mediator HMGB1, representing a more clinically feasible target compared to the early-peaking cytokines that limited previous trials. Its selective inhibition of the pathological HMGB1-pCTS-L axis further offers a nuanced immunomodulatory approach that spares beneficial host inflammatory responses. For RA, P2-1 addresses key limitations of current anti-TNF biologics, notably systemic immunosuppression and treatment non-response. By targeting a novel downstream pathway, it offers a new therapeutic option for refractory patients, while its “pathology-activated” mechanism provides a superior safety profile compared to existing systemic immunosuppressants. Therefore, P2-1 concurrently emerges as a highly promising next-generation therapeutic for RA and a more targeted intervention for sepsis, underscoring its potential for broad clinical utility.

However, the successful clinical translation of P2-1 necessitates addressing two key preclinical challenges: PK and immunogenicity. For the P2-1 peptide, this involves optimizing its half-life using established technologies like PEGylation, guided by multi-species PK studies. While immunogenicity is an inherent risk, it is substantially mitigated by the high conservation of its target (HMGB1, sharing 98–99% sequence identity between rodents and humans) and the peptide’s demonstrated activity on human cells. A standard risk assessment, including in silico screening and ex vivo T-cell assays, will help determine the best approach to de-immunize the peptide if necessary. For the anti-pCTS-L mAbs, the crucial first step is humanization to minimize their immunogenicity before human application. Following these preclinical optimizations, the clinical development pathway will then progress from Phase I safety trials in healthy volunteers to Phase II proof-of-concept studies in patients who exhibit elevated levels of HMGB1 and pCTS-L biomarkers.

Despite these promising findings, certain limitations warrant discussion. Our reliance on preclinical animal models of sepsis and RA necessitates careful consideration of species-specific differences in immune responses during clinical translation. While we elucidated a key mechanistic pathway involving P2-1, HMGB1, and pCTS-L, the full spectrum of P2-1’s interactions and downstream effects requires further exploration. The selective suppression of HMGB1-induced *Ctsl* mRNA upregulation by P2-1, without broadly reversing other HMGB1-induced cytokines/chemokines, highlights its specificity but also calls for deeper investigation into its precise targets. Similarly, it is critically important to systemically investigate the effect of P2-1 and anti-pCTS-L mAbs on the multi-faceted process of joint bone destruction and autoantibody production in a more clinically relevant collagen-induced arthritis model of human RA. Finally, future work will need to address the long-term safety, PK, and PD of P2-1 in larger animal models and ultimately, in human clinical trials.

## Conclusions

In conclusion, our study reveals a paradigm-shifting drug discovery strategy that transforms counterintuitive insights from a detrimental antibody into powerful and targeted therapeutics. This approach yields P2-1 as a novel therapy that confers significant protection against both sepsis and RA, even when it is administered therapeutically after disease onset. Mechanistically, P2-1 binds HMGB1 to selectively suppress HMGB1-induced *Ctsl* mRNA upregulation and pCTS-L secretion, thereby effectively disrupting the crucial HMGB1-pCTS-L inflammatory axis. Furthermore, our findings establish pCTS-L as a key mediator of inflammatory diseases, validating it as a novel therapeutic target for RA. These findings open exciting new avenues for drug development, underscoring the potential of precisely modulating harmful protein–protein interactions (via decoy peptides or neutralizing antibodies) to achieve therapeutic outcomes in complex inflammatory disorders.

## Supplementary Information

**Additional file 1.** **Fig. S1** Modulation of HMGB1 extracellular functions through distinct molecular interactions. **Fig. S2** Two representative full-range surface plasmon resonance (SPR) plots demonstrating the interaction between HMGB1 and P2-1. **Fig. S3** Predicted P2 peptide structure and its docking interaction with HMGB1 B-box. **Fig. 4** A tetranectin (TN) mutant lacking the N-terminal α-helix trimerization domain retained the protective efficacy of TN in sepsis. Fig. S5 Representative Cytokine Antibody Arrays depicting the effect of P2-1 on collagen antibody-induced arthritis (CAIA)-induced joint inflammation. **Fig. S6** Prophylactic P2-1 treatment attenuated collagen antibody-induced arthritis (CAIA). **Fig. S7** Volcano plots illustrating P2-1-modulated differential gene expression in HMGB1-stimulated human PBMCs. **Fig. S8** Representative cytokine antibody arrays illustrating the effects of P2-1 on HMGB1-induced cytokines and chemokines. **Fig. S9** Full Western blotting analysis of the effect of P2-1 on HMGB1-induced pCTS-L release in human PBMCs. **Fig. S10** Representative cytokine antibody arrays illustrating the effect of mAb2 on collagen antibody-induced arthritis (CAIA)-induced joint inflammation. **Fig. S11** Proposed model for P2-1-mediated intervention in the inflammatory HMGB1-pCTS-L axis in sepsis and rheumatoid arthritis. **Table S1** Key reagent sources

**Additional file 2.** PDB files for the predicted P2 structure

**Additional file 3.** PDB files for the docking model of the HMGB1 B-box/P2 complex

**Additional file 4.** Primary data

## Data Availability

The anti-pCTS-L mAb2 can be provided by Dr. Hai-Chao Wang pending scientific review and a completed material transfer agreement. All data needed to evaluate the conclusions in the paper are present in the paper and the Additional files.

## Abbreviations

ΔTN: Tetranectin N-terminal deletion mutant
3D: Three-dimensional
ACS: Antigen contact structure
BLC: B-lymphocyte chemoattractant
CAIA: Collagen antibody-induced arthritis
CLP: Cecal ligation and puncture
CRD: Carbohydrate recognition domain
Ctsl: Cathepsin L
DAMPs: Damage-associated molecular patterns
HMGB1: High mobility group box 1
IL: Interleukins
i.p.: Intraperitoneal
IQR: Interquartile range
GRO: Growth-regulated oncogene
KC: Keratinocyte-derived chemokine
LDH: Lactate dehydrogenase
LPS: Lipopolysaccharides
mAb: Monoclonal antibody
MCP-1: Monocyte chemoattractant protein-1
MIP-1γ: Macrophage inflammatory protein-1γ
PBMCs: Human peripheral blood mononuclear cells
pCTS-L: Procathepsin L
PD: Pharmacodynamics
PK: Pharmacokinetics
RA: Rheumatoid arthritis
RAGE: Receptor for advanced glycation end products
RNA-Seq: RNA sequencing
SPR: Surface plasmon resonance
sTNFR: Soluble tumor necrosis factor receptor
TN: Tetranectin
TNF: Tumor necrosis factor
VCAM-1: Vascular cell adhesion molecule-1
