# Supplementary material for: Repurposing a detrimental antibody epitope as targeted therapeutics for sepsis and rheumatoid arthritis
Source: Mil Med Res. 2026 Feb 17;12:98. doi: 10.1186/s40779-026-00686-8 (PMC12914960; doi:10.1186/s40779-026-00686-8)
Supplement: Supplementary file 1 — Additional file 1. Fig. S1 Modulation of HMGB1 extracellular functions through distinct molecular interactions. Fig. S2 Two representative full-range surface plasmon resonance (SPR) plots demonstrating the interaction between HMGB1 and P2-1. Fig. S3 Predicted P2 peptide structure and its docking interaction with HMGB1 B-box. Fig. 4 A tetranectin (TN) mutant lacking the N-terminal α-helix trimerization domain retained the protective efficacy of TN in sepsis. Fig. S5 Representative Cytokine Antibody Arrays depicting the effect of P2-1 on collagen antibody-induced arthritis (CAIA)-induced joint inflammation. Fig. S6 Prophylactic P2-1 treatment attenuated collagen antibody-induced arthritis (CAIA). Fig. S7 Volcano plots illustrating P2-1-modulated differential gene expression in HMGB1-stimulated human PBMCs. Fig. S8 Representative cytokine antibody arrays illustrating the effects of P2-1 on HMGB1-induced cytokines and chemokines. Fig. S9 Full Western blotting analysis of the effect of P2-1 on HMGB1-induced pCTS-L release in human PBMCs. Fig. S10 Representative cytokine antibody arrays illustrating the effect of mAb2 on collagen antibody-induced arthritis (CAIA)-induced joint inflammation. Fig. S11 Proposed model for P2-1-mediated intervention in the inflammatory HMGB1-pCTS-L axis in sepsis and rheumatoid arthritis. Table S1 Key reagent sources [file 40779_2026_686_MOESM1_ESM.pdf]

## Methods

### Study design

For these preclinical studies, animals were randomly assigned to experimental groups and treated with P2, P2-1, or a pCTS-L-neutralizing mAb2 according to the indicated dosing regimens. Outcomes, including septic survival rates, arthritis severity, pain sensitivity, and joint tissue histology scores, were collected under blinded experimental conditions. The study design and sample sizes for each experiment are detailed in the figure legends. No data, including outlier values, were excluded. For skewed data, outliers were defined via the interquartile range (IQR) method as any value below $Q_1 - (1.5 \times \text{IQR})$ or above $Q_3 + (1.5 \times \text{IQR})$. For normally distributed data, outliers were defined via the Z-score method as any value with an absolute Z-score greater than 2 or 3. The primary data are reported in **Additional file 4**. All the reagent sources are listed in **Additional file 1: Table S1**.

### Cecal ligation and puncture (CLP) sepsis model

BALB/c mice were anesthetized intraperitoneally with ketamine (87.5 mg/kg, Henry Schein Animal Health, Dublin, OH, USA) and xylazine (12.5 mg/kg, Sigma-Aldrich, St. Louis, MO, USA) before a midline abdominal incision was made in the lower left abdomen. The cecum was ligated with a 4-0 silk suture at 50% of its length from the distal end, and punctured once with a 22-gauge needle to induce moderate to severe sepsis. The cecum was returned to the abdominal cavity, and the incision was closed in layers. Approximately 30 min post-CLP, animals received imipenem/cilastatin (0.5 mg/mouse, Primaxin, Merck & Co., Inc., Rahway, NJ, USA) and 1.0 ml of sterile saline for fluid resuscitation. We chose i.p. injection primarily to establish initial proof-of-concept for systemic efficacy, and to determine whether a systemically delivered peptide or protein could reach and effectively attenuate inflammation in sepsis.

### Collagen antibody-induced arthritis (CAIA) model

On Day 0, the mice received an i.p. injection of 2 mg of anti-collagen II mAb cocktail (α-CII, Chondrex, Inc., Redmond, WA). On Day 3, the mice received an i.p. injection of 30 μg of LPS [*Escherichia coli* (*E. coli*) O111:B4; Sigma-Aldrich] to activate inflammation and synchronize arthritis onset. Therapeutic agents [P2-1 peptide (1.0 or 2.0 mg/kg) or pCTS-L-neutralizing mAb2 (1.0, 2.0, or 4.0 mg/kg)] were administered i.p. daily, either prophylactically from – Day 2 or therapeutically from Day 6 post-α-CII challenge. Although intra-articular injection represents a clinically relevant route for RA treatment, it was deliberately avoided in this initial study to preclude confounding inflammation that could arise from the injection trauma itself within the small mouse joint. We selected i.p. injection primarily to establish initial proof-of-concept for systemic efficacy and to determine whether a systemically delivered peptide or antibody could reach and effectively attenuate joint inflammation in CAIA-induced arthritis. The demonstrated success of systemic delivery in this initial study will provide a strong rationale to evaluate clinically relevant systemic routes [e.g., subcutaneously (SC) or intravenously (IV)] in future studies.

**Protein expression, purification, and peptide synthesis**

Recombinant CBP-tagged HMGB1 was purified via calmodulin-affinity chromatography, and endotoxins were removed from the HMGB1 preparation via Triton X-114 extractions. A recombinant ΔTN, lacking residues 1 – 44 (heparin binding and part of the α-helix trimerization domain), was expressed in DE3 pLysS with an N-histidine tag, and was purified to homogeneity following a similar protocol to that for full-length TN [1]. Briefly, after sonication to disrupt bacteria, ΔTN inclusion bodies were isolated by differential centrifugation following extensive washing in 1× PBS containing 1% Triton X-100. The inclusion bodies were then solubilized in 8 mol/L urea, and refolded by dialysis in 10 mmol/L Tris buffer (pH = 8.0) containing N-lauroylsarcosine. The recombinant ΔTN was subsequently subjected to extensive Triton X-114 extractions to remove contaminating endotoxins. The P2 peptide (55-KVHMKCFLAFQTKTF-70; Lot # U114VHD110-1/PE6854; Purity, 98.1%) and its water-soluble derivative P2-1 [54-TKVH(Nle)KSFLAFQTKT-69; Lot # U114VHD110-4/PE6857; Purity, 99.6%] were

custom-synthesized (GenScript, Piscataway, NJ, USA). P2-1 was engineered to: i) increase solubility by removing a water-insoluble phenylalanine (F) at the C-terminal residue 70 and introducing a water-soluble threonine (T) at N-terminal residue 54; and ii) reduce oxidative susceptibility by substituting methionine (M) with norleucine (Nle) and cysteine (C) with serine (S), respectively. The pCTS-L-neutralizing mAbs (mAb2) and TN-neutralizing mAbs (mAb8 and mAb9) were generated in BALB/c and C57BL/6 mice as previously described [1, 2].

**RNA sequencing (RNA-Seq) and bioinformatics analysis**

Total RNA was isolated from human PBMCs (*n* = 6 biological replicates) stimulated with HMGB1 in the absence or presence of P2-1 (5.0 or 10.0 μg/ml) via the RNeasy Mini Kit (Qiagen, Germantown, MD, USA) according to the manufacturer's instructions. RNA quantity and quality were assessed via a NanoDrop spectrophotometer, which requires A260/280 and A260/230 ratios to both be > 1.8. Samples meeting these criteria were then sent to two sequencing facilities, where RNA integrity was further confirmed via an Agilent Bioanalyzer. Only samples with an RNA integrity number of 8 or above were accepted for RNA sequencing. cDNA libraries were prepared using the TruSeq RNA Library Prep Kit v2 (Illumina, San Diego, CA, USA) and sequenced on an Illumina NovaSeq 6000 platform to generate 100 bp paired-end reads. Our data filtering criteria adhered to industry best practices to ensure data quality. First, raw sequencing reads underwent quality assessment with FastQC, followed by adapter and low-quality nucleotide trimming using Trimmomatic v.0.36. Subsequently, the high-quality reads were aligned to the Homo sapiens GRCh38 reference genome using the STAR aligner v.2.5.2b. Following alignment, PCR duplicates were identified and removed with Picard tools to mitigate amplification bias. Before differential expression analysis, the gene count matrix was filtered to retain only genes with a count-per-million (CPM) > 1 in at least three samples (*n* = 3, representing our smallest experimental group). This rigorous procedure ensured that analysis was performed on a robust set of reliably expressed genes. Finally, gene counts were quantified via StringTie and DESeq2 for differential gene expression analysis. Volcano

plots and heatmaps were generated via ggplot2 and pheatmap packages in R, respectively. Genes with an adjusted *P*-value < 0.05 and a $|\log_2 \text{fold change}| > 1$ were considered differentially expressed.

**Human macrophage differentiation and treatment**

Human PBMCs were differentiated into macrophages by culturing in human macrophage colony-stimulating factor (M-CSF, 20 ng/ml) for 5 – 6 d. Differentiated macrophages were maintained in Dulbecco's modified Eagle's medium supplemented with 1% penicillin/streptomycin and 10% human serum. Upon reaching 70 – 80% confluence, adherent cells were washed and switched to Opti-MEM I before stimulation with recombinant HMGB1 (0.5 μg/ml) with or without P2-1 (5.0 or 10 μg/ml) for 16 h.

**Cell viability**

Cell viability was assessed via the trypan blue exclusion and lactate dehydrogenase (LDH) release method as previously described [1]. For trypan blue exclusion, random phase-contrast images were captured, and the percentage of nonviable, trypan blue-stained cells was calculated. LDH release in the culture medium was quantified with an LDH Assay Kit (Cat. #L7572, Pointe Scientific Inc., Canton, MI, USA) according to the manufacturer's instructions. Optical density was measured at 340 nm, and LDH content was expressed as a percentage of maximal release in the presence of 2% Triton X-100. The experiments were performed 5 – 7 times (*n* = 5 – 7 biological replicates).

**Cellular uptake of HMGB1**

Highly purified recombinant HMGB1 was labeled with Alexa Fluor 555 via a commercial kit (Cat. #A30007, Thermo Fisher Scientific, Waltham, MA, USA) according to the manufacturer's instructions. Differentiated human macrophages, cultured on coverslips, were incubated with Alexa Fluor 555-labeled HMGB1 (0.5 μg/ml) alone, or in the presence of P2-1 (10.0 μg/ml) and the endocytosis inhibitor, dynasore (8.0 μmol/L) at 37 °C for 2 h. Following incubation, the cells were extensively washed with 1× PBS to

remove unbound protein and then fixed with 4% paraformaldehyde for 20 min at room temperature. Coverslips were mounted onto slides via Vectashield Mounting Medium for fluorescence with DAPI (Cat. #H-1200, Vector Laboratories, Inc., Newark, CA, USA) for nuclear counterstaining. Endocytic uptake of Alexa Fluor 555-labeled HMGB1 (red) was visualized via the Olympus IX51 inverted fluorescence & phase contrast tissue culture microscope. Cellular HMGB1 uptake was quantified by measuring the mean fluorescence intensity of the Alexa-555-labeled HMGB1 protein within multiple representative cells via ImageJ analysis software.

**Antibody sequencing and antigen contact structure (ACS) prediction**

Total RNA was extracted from mAb8 and mAb9 hybridoma cells via TRIzol (Invitrogen, Austin, TX, USA), and cDNA was synthesized via reverse transcriptase. Variable regions of heavy and light chains were amplified via PCR using degenerate primers and sequenced [1]. CDR sequences were identified via the Kabat numbering scheme. Computational modeling of the mAb-antigen interaction was performed via ABodyBuilder-ML (SAbPred). The predicted three-dimensional (3D) structures of the antibody paratopes allowed visualization of the ACS.

**Epitope mapping via dot blotting**

Corresponding to the C-terminal portion of the TN protein (**Additional file 1: Fig. S4a**), a library of 10 synthetic peptides (P1 – P10, **Additional file 1: Fig. S4b**) was spotted (0.1 µg in 2.5 µl) onto nitrocellulose membranes (Cat No. 88013, Thermo Scientific, Waltham, MA, USA). The membranes were blocked with 5% milk in PBS-T (PBS with 0.1% Tween-20) and then incubated with anti-TN monoclonal antibodies (mAb8 or mAb9, 1.0 µg/ml). After washing, the membranes were incubated with HRP-conjugated goat anti-mouse IgG (Jackson ImmunoResearch, West Grove, PA, USA), and signals were detected via chemiluminescence as previously described [1].

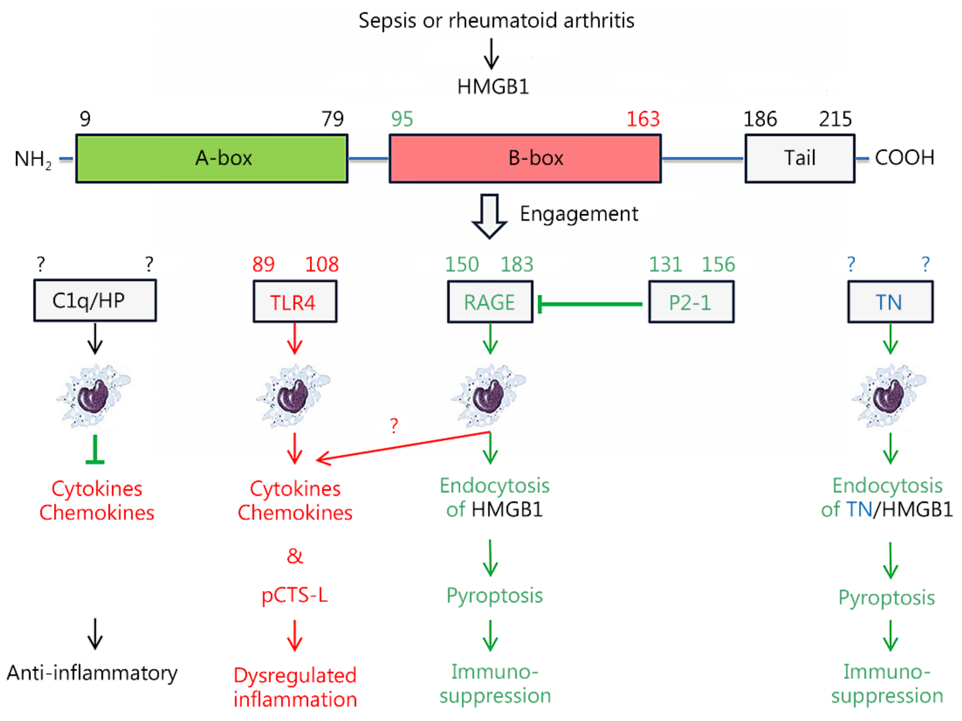


**Fig. S1** Modulation of HMGB1 extracellular functions through distinct molecular interactions. The top panel illustrates HMGB1's domain structure, comprising an anti-inflammatory "A-box", a proinflammatory "B-box", and a C-terminal acidic tail. The bottom panel depicts the divergent functional outcomes stemming from HMGB1's interaction with different proteins. For example, HMGB1 directly engages pattern recognition receptors such as TLR4 (via residues 89 – 108) and RAGE (via residues 150 – 183), leading to innate immune activation and macrophage pyroptosis, respectively. Conversely, endogenous proteins such as complement component 1q (C1q) and haptoglobin (HP) bind to HMGB1 at unknown residues, inhibiting its proinflammatory effects through distinct signaling pathways. Notably, tetranectin (TN) interacts with HMGB1 to increase HMGB1-induced pyroptosis by facilitating the endocytosis of HMGB1/TN complexes through unknown TN receptors. In contrast, P2-1 functions as an "off-switch" by binding HMGB1 to prevent its interaction with pro-endocytic receptors (e.g., RAGE), thereby suppressing HMGB1 uptake, macrophage pyroptosis, and HMGB1-mediated pCTS-L upregulation. However, it remains unknown ("?") whether HMGB1 induces pCTS-L upregulation through

RAGE or other unknown receptors. HMGB1 high mobility group box 1, TLR4 Toll-like receptor 4, RAGE receptor for advanced glycation products, pCTS-L procathepsin L

a

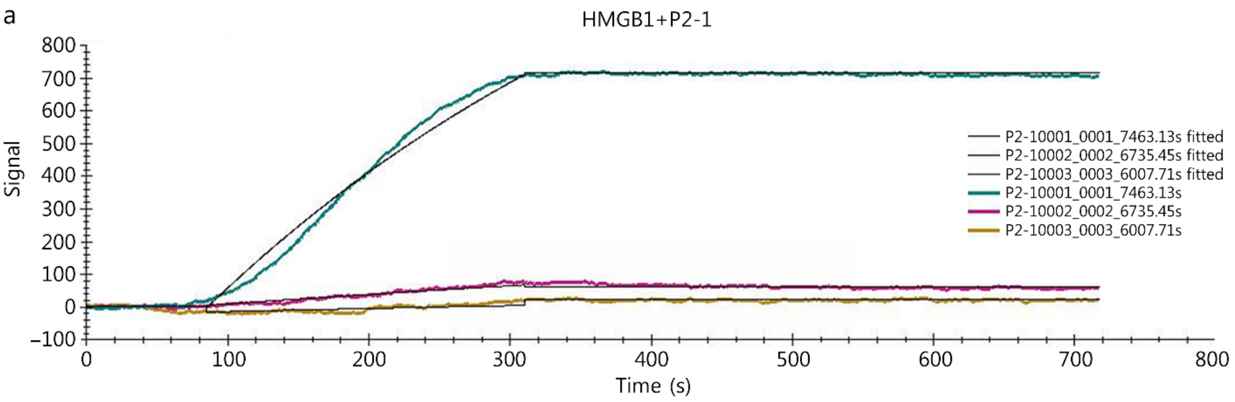


Model fit lines (Black)

Experimental data lines (Color)

| Curve name | Bmax (Signal) | $k_a$ [1/(M*s)] | $k_d$ (1/s) | $K_D$ (M) | BI (Signal) | $Chi^2$ ($Signal^2$) | *U*-value: $k_d$ (%) |
|---|---|---|---|---|---|---|---|
| P2-1 01_7463.13s fitted | 1626.53 | 22.4 | $5.22\times10^{-8}$ | $2.33\times10^{-9}$ | −4.06 | 130.52 | >50 |
| P2-1 02_6735.45s fitted | 354.12 | 22.4 | $5.22\times10^{-8}$ | $2.33\times10^{-9}$ | 4.12 | 130.52 | >50 |
| P2-1 03_6007.71s fitted | 325.91 | 22.4 | $5.22\times10^{-8}$ | $2.33\times10^{-9}$ | −16.37 | 130.52 | >50 |

b

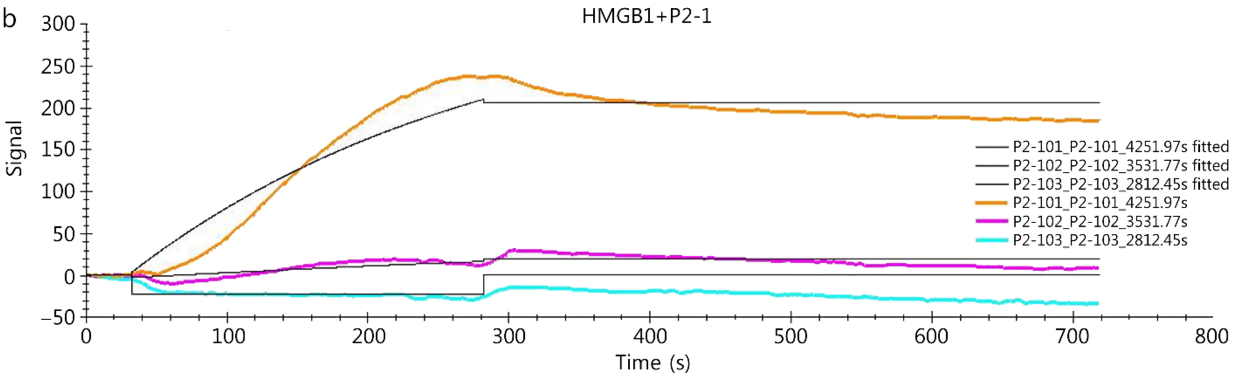


Model fit lines (Black)

Experimental data lines (Color)

| Curve name | Bmax (Signal) | $k_a$ [1/(M*s)] | $k_d$ (1/s) | $K_D$ (M) | BI (Signal) | $Chi^2$ ($Signal^2$) | *U*-value: $k_d$ (%) |
|---|---|---|---|---|---|---|---|
| P2-1 01_4251.97s fitted | 330.06 | 68.7 | $2.30\times10^{-6}$ | $3.35\times10^{-8}$ | 3.71 | 279.31 | >50 |
| P2-1 02_3531.77s fitted | 70.27 | 68.7 | $2.30\times10^{-6}$ | $3.35\times10^{-8}$ | −3.20 | 279.31 | >50 |
| P2-1 03_2812.45s fitted | 0 | 68.7 | $2.30\times10^{-6}$ | $3.35\times10^{-8}$ | −23.17 | 279.31 | >50 |

**Fig. S2** Two representative full-range surface plasmon resonance (SPR) plots demonstrating the interaction between high mobility group box 1 (HMGB1) and P2-1. SPR plots showing the interaction between immobilized HMGB1 and different concentrations of the P2-1 analyte. Experimental binding responses (blue, purple, and yellow traces) are shown with their corresponding global fits (black curves) via a 1:1 interaction model. The associated table summarizes the globally fitted kinetic parameters, including the maximum binding capacity (Bmax), association rate constant ($k_a$), dissociation rate constant ($k_d$), and other goodness-of-fit metrics ($Chi^2$). The relatively low $Chi^2$ value indicated a good fit between

the model and the experimental data. By dividing the dissociation rate ($k_d$) by the association rate ($k_a$), the equilibrium dissociation constant ($K_D$) was calculated as a measure of the affinity between HMGB1-P2-1 interactants

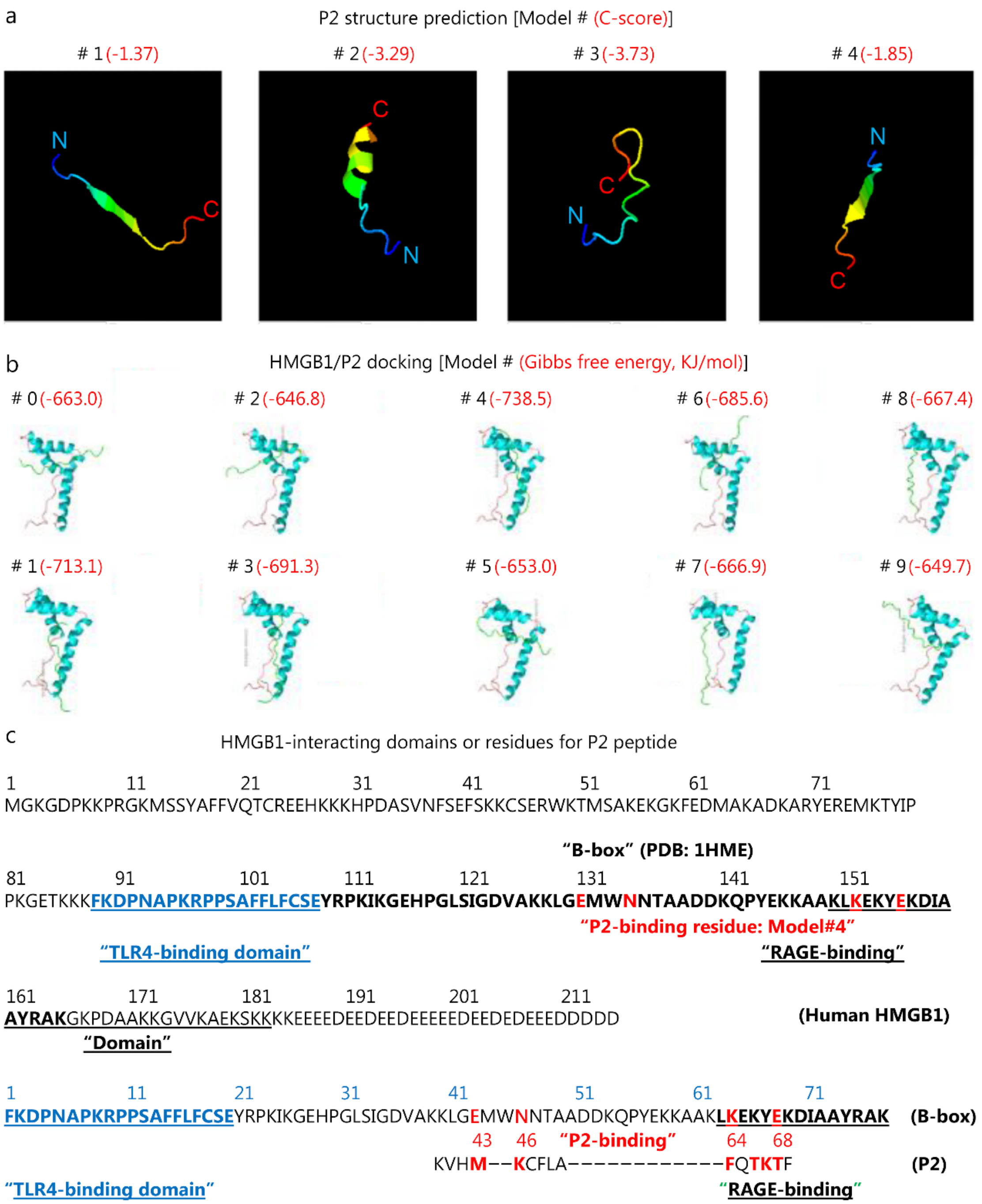


**Fig. S3** Predicted P2 peptide structure and its docking interaction with the HMGB1 B-box. **a** Predicted 3D structure of the P2 peptide. The models were generated via the I-TASSER (Iterative Threading ASSembly Refinement) web server (https://zhanggroup.org/I-TASSER/). Model confidence is indicated by its C-score, where higher values denote greater confidence and better model quality. **b** ClusPro protein-protein docking of HMGB1 B-box and P2. The interaction between the human HMGB1 B-box (PDB:

1HME) and the P2 peptide was modeled via the ClusPro web server (https://cluspro.bu.edu/login.php). The docking models are ranked by their estimated binding free energy (Gibbs free energy), with lower values signifying higher confidence. **c** Interfacial residues in the HMGB1/P2 complex. Key interacting residues within the HMGB1 and B-box sequences are highlighted in red. The P2-binding residues are distant from its TLR4-binding domain (residues 89 – 108) but partially overlap with the RAGE-binding domain (residues 150 – 183, including E153 and K154). This spatial arrangement suggests that P2 or P2-1 may selectively interfere with RAGE-dependent HMGB1 activities (e.g., endocytosis and macrophage pyroptosis induction) without affecting TLR4-dependent functions (e.g., the induction of cytokines and chemokines). HMGB high mobility group box 1, 3D three-dimensional, PDB protein data bank, TLR toll-like receptor, RAGE receptor for advanced glycation end products

a

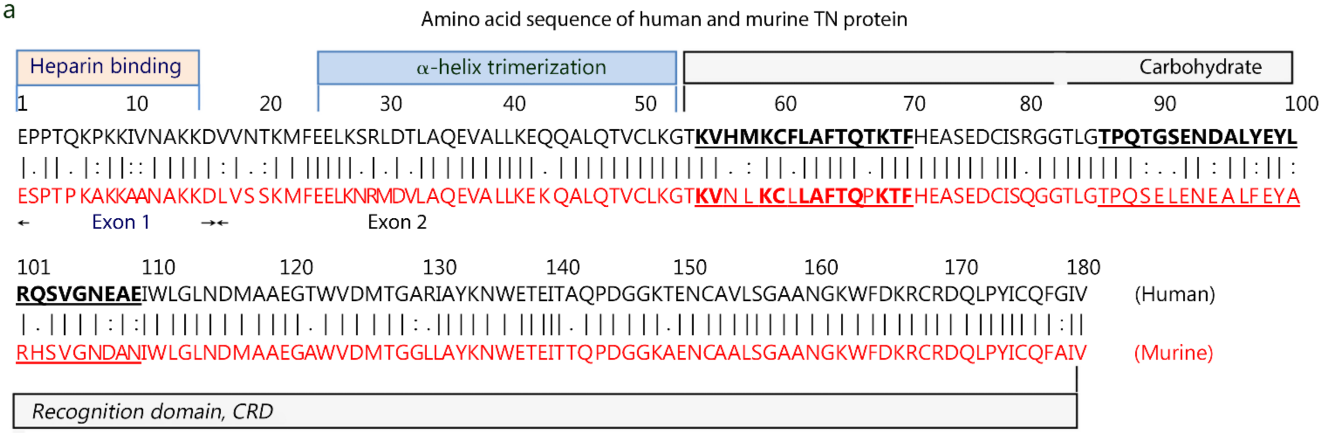


b Sequence of synthetic peptides

| Peptide # | Sequence |
|---|---|
| P1 | 46-LQTVCLKGT-54 |
| P2 | 55-KVHMKCFLAFTQTKTF-70 |
| P3 | 71-HEASEDCISRGG-83 |
| P4 | 81-GGTLGTPQTG-90 |
| P5 | 86-TPQTGSENDALYEYLRQSVGNEAE-109 |
| P6 | 105-GNEAEIWLGLNDMAAEGT-122 |
| P7 | 121-GTWVDMTGARIAYKNWETEITAQP-144 |
| P8 | 140-ITAQPDGGKTENC-152 |
| P9 | 151-NCAVLSGAANGKWFDKR-167 |
| P10 | 158-AANGKWFDKRCRDQLPYICQFGIV-181 |

c

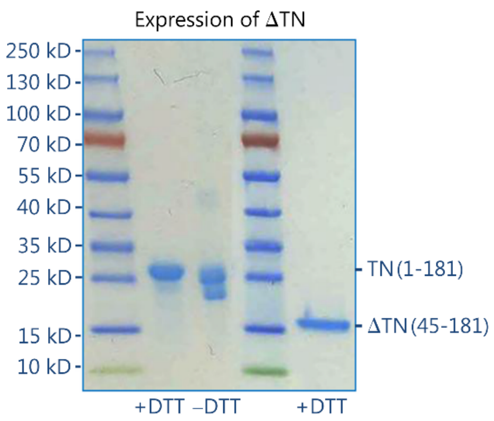


d

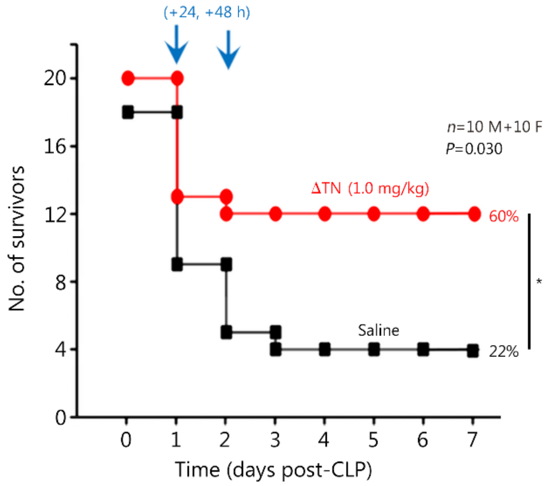


**Fig. S4** A tetranectin (TN) mutant lacking the N-terminal α-helix trimerization domain retained the protective efficacy of TN in sepsis. **a** Domain architecture of human and murine TNs, beginning after the N-terminal 21-amino acid leader signal sequence. **b** Sequences of 10 synthetic peptides derived from the human TN carbohydrate recognition domain (CRD), including the P2 epitope, used for functional

screening. **c** Expression and purification of recombinant full-length human TN (residues 1 – 181) and its N-terminal deletion mutant (ΔTN, residues 45 – 181). Both constructs with an N-histidine tag were expressed in *E. coli* BL21 (DE3) pLysS cells as insoluble inclusion bodies, which were isolated by differential centrifugation following extensive washing in 1× PBS containing 1% Triton X-100. The inclusion bodies were then solubilized in 8 mol/L urea, and refolded by dialysis in 10 mmol/L Tris buffer (pH 8.0) containing N-lauroylsarcosine. Subsequently, recombinant ΔTN was subjected to extensive Triton X-114 extractions to remove contaminating endotoxins. **d** Recombinant ΔTN conferred significant protection against lethal sepsis. Male ($n$ = 10) and female ($n$ = 10) BALB/c mice were subjected to cecal ligation and puncture (CLP). Recombinant ΔTN was given intraperitoneally at 24 h and 48 h post-CLP, and animal survival rates were monitored. $^{*}P < 0.05$. *E. coli Escherichia coli*, ΔTN tetranectin N-terminal deletion mutant, DTT dithiothreitol

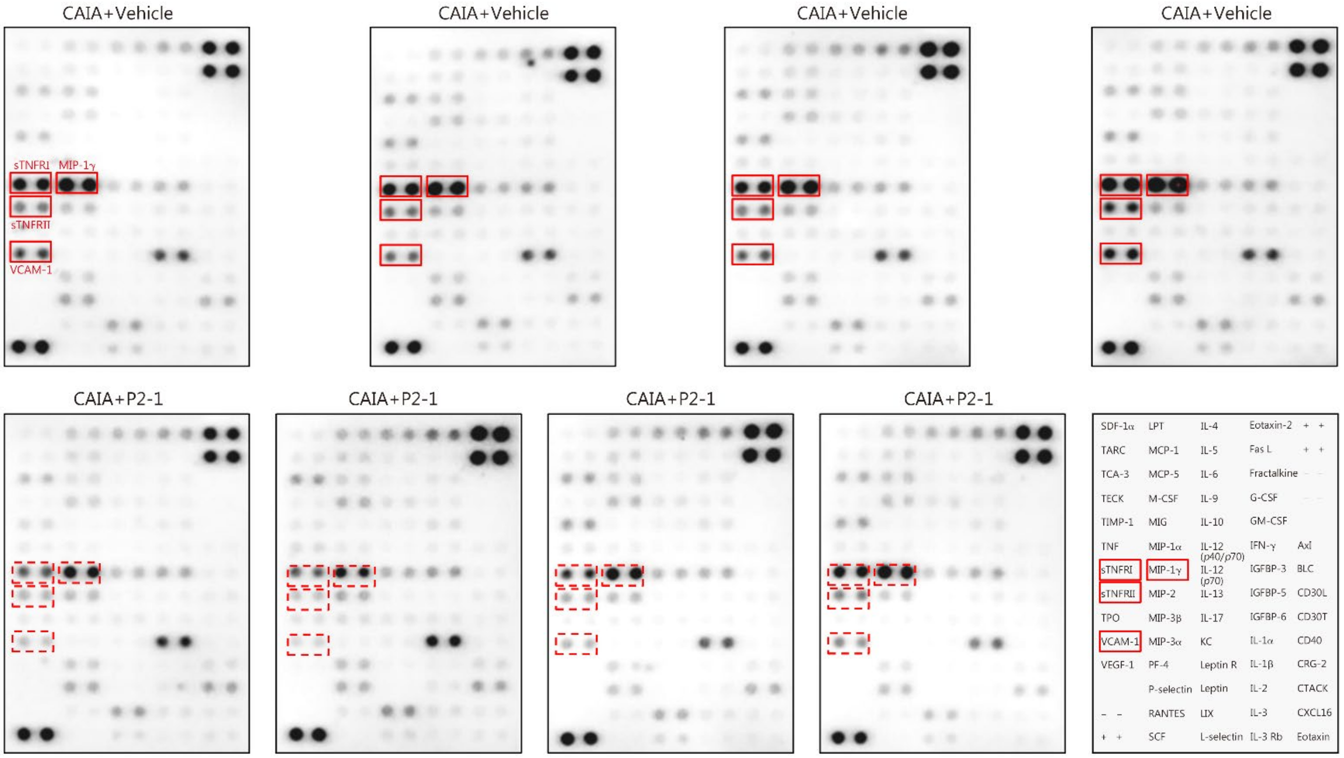


**Fig. S5** Representative cytokine antibody arrays depicting the effect of P2-1 on collagen antibody-induced arthritis (CAIA)-induced joint inflammation. Representative images from cytokine antibody arrays of joint tissue lysates from 4 vehicle-treated CAIA mice and 4 P2-1-treated CAIA mice (harvested on Day 10). These findings revealed a visible reduction in various inflammatory cytokines and chemokines in P2-1-treated samples, supporting the quantitative data in **Fig. 4d**. sTNFR soluble tumor necrosis factor receptor, MIP macrophage inflammatory protein, VCAM vascular cell adhesion molecule, VEGF vascular endothelial growth factor, PF platelet factor, SDF stromal-derived factor, TARC thymus and activation-regulated chemokine, TECK thymus-expressed chemokine, TIMP tissue inhibitor of metalloproteinases, TNF tumor necrosis factor, TPO thrombopoietin, MCP monocyte chemoattractant protein, M-CSF macrophage colony-stimulating factor, MIG monokine induced by gamma interferon, RANTES regulated on activation normal T cell expressed and secreted, SCF stem cell factor, FasL fas ligand, G-CSF granulocyte colony-stimulating factor, GM-CSF granulocyte macrophage colony-stimulating factor, IFN interferon, IGFBP insulin-like growth factor binding protein, IL interleukin, Axl receptor tyrosine kinase,

BCL B-lymphocyte chemoattractant, CD30L CD30 ligand, CRG cytokine-responsive gene, CTACK cutaneous T lymphocyte-attracting chemokine, CXCL C-X-C motif chemokine ligand

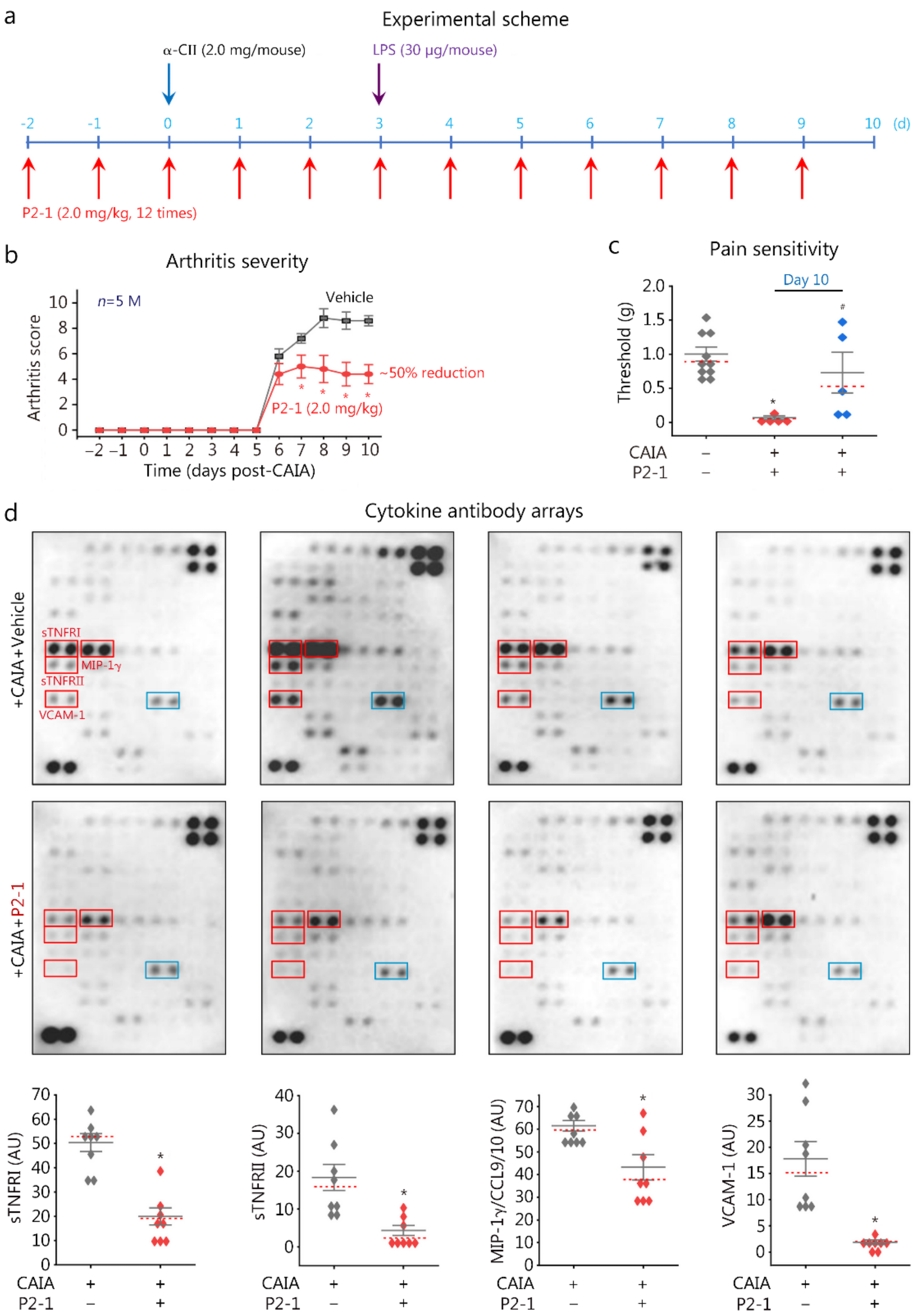


**Fig. S6** Prophylactic P2-1 treatment attenuated collagen antibody-induced arthritis (CAIA). **a** Schematic of the CAIA model and prophylactic P2-1 treatment regimen. **b** P2-1 treatment significantly reduced the severity of CAIA-induced arthritis. $^{*}P$ < 0.05 vs. the vehicle group, non-parametric Kruskal-Wallis ANOVA test. **c** P2-1 treatment significantly reversed CAIA-induced mechanical hypersensitivity, as measured by paw mechanical withdrawal thresholds on Day 10 post-CAIA. $^{*}P$ < 0.05 vs. the nonarthritic

negative control ("- CAIA"); $^{\#}P < 0.05$ vs. the vehicle-treated positive control ("+ CAIA") on the same day. **d** P2-1 treatment significantly reduced joint levels of key inflammatory mediators (sTNFRI/II, MIP-1γ, and VCAM-1) on Day 10. IL-1α served as a negative control. $^{*}P < 0.05$ vs. the vehicle-treated control ("+ CAIA"), non-parametric Kruskal-Wallis ANOVA test. LPS lipopolysaccharides, sTNFR soluble tumor necrosis factor receptor, MIP-1 monocyte chemoattractant protein-1, VCAM-1 vascular cell adhesion molecule-1

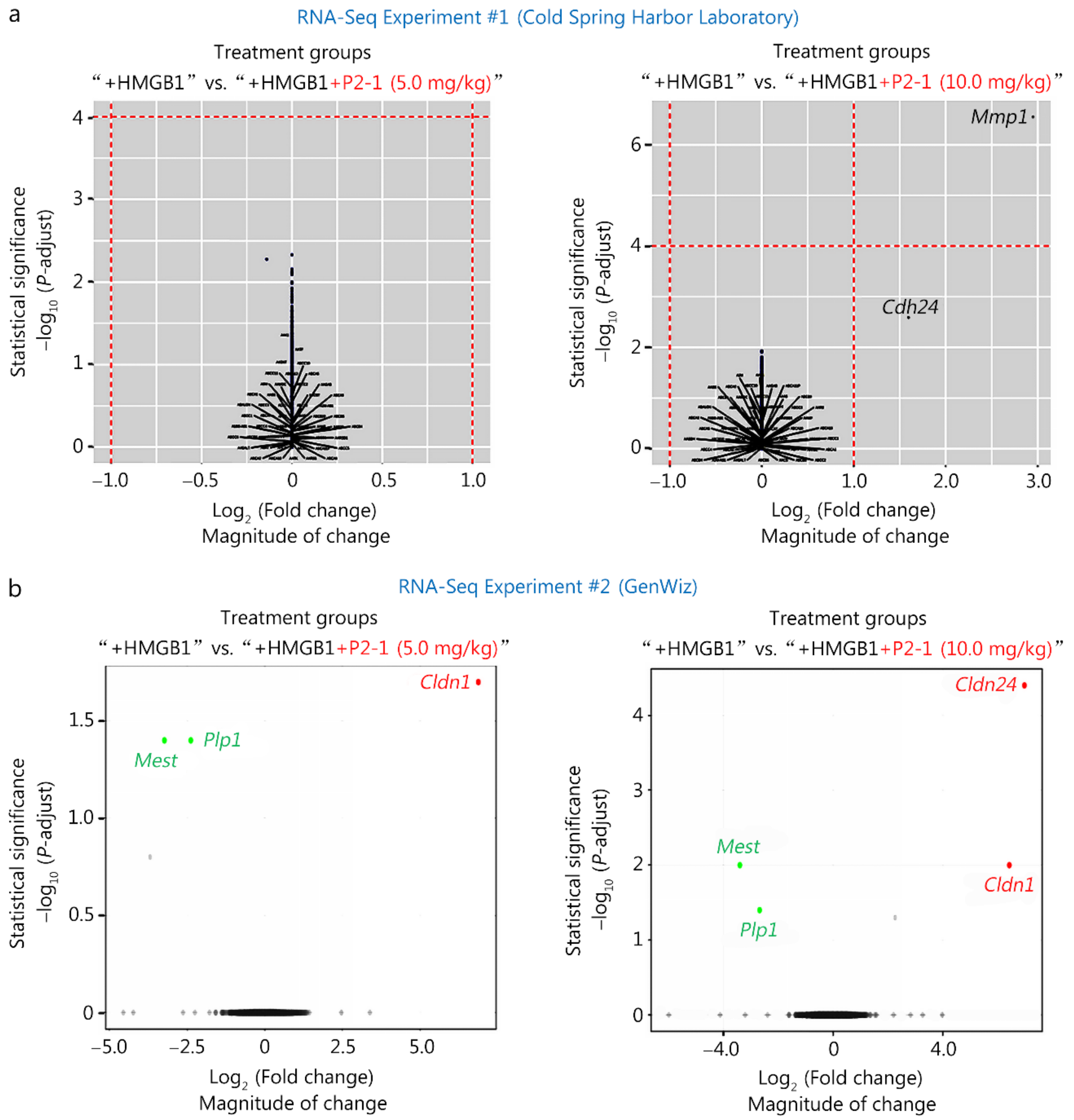


**Fig. S7** Volcano plots illustrating P2-1-modulated differential gene expression in HMGB1-stimulated human PBMCs. Data were generated from two distinct RNA-seq studies performed at Cold Spring Harbor Laboratory and GenWiz. Volcano plots were used to identify genes significantly up- or down-regulated by P2-1 in human PBMCs stimulated with HMGB1. The X-axis represents the $\log_2$ fold change in gene expression between PBMCs treated with HMGB1 alone and those treated with HMGB1 + P2-1 at 5.0 µg/ml (top panel) or 10.0 µg/ml (bottom panel). The Y-axis represents the statistical significance as the -$\log_{10}$ (adjusted *P*-value) for the observed expression change in each gene. The observed tight clustering of most data points around a 0 $\log_2$ fold change and near-zero -$\log_{10}$ (adjusted *P*-value) implies that P2-1

does not induce widespread reversal of HMGB1-orchestrated transcriptional changes. HMGB high mobility group box 1, RNA-Seq RNA sequencing, PBMCs peripheral blood mononuclear cells

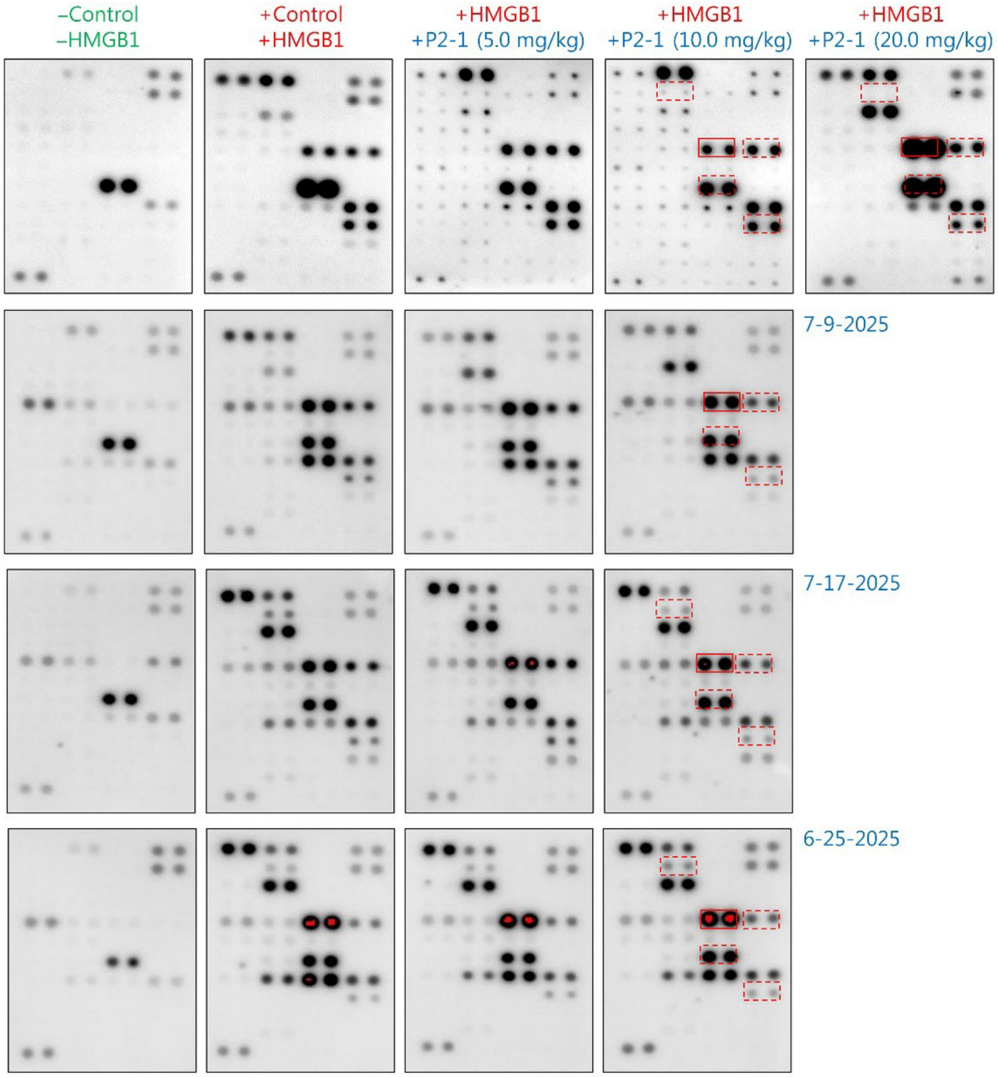


**Fig. S8** Representative cytokine antibody arrays illustrating the effects of P2-1 on HMGB1-induced cytokines and chemokines. Human PBMCs were stimulated with HMGB1 (0.5 mg/ml) in the absence or presence of P2-1 (5.0, 10.0, or 20.0 μg/ml) for 16 h, and the extracellular levels of cytokines and chemokines were determined via cytokine antibody arrays. The experiments were conducted on June 25th, July 9th, and July 17th, 2025 (6-25-2025, 7-9-2025, and 7-17-2025). Comprehensive cytokine antibody array results revealed that P2-1 (5.0 and 10.0 μg/ml) does not broadly inhibit the HMGB1-induced secretion of most cytokines and chemokines from human PBMCs. HMGB1 high mobility group box 1, PBMCs peripheral blood mononuclear cells

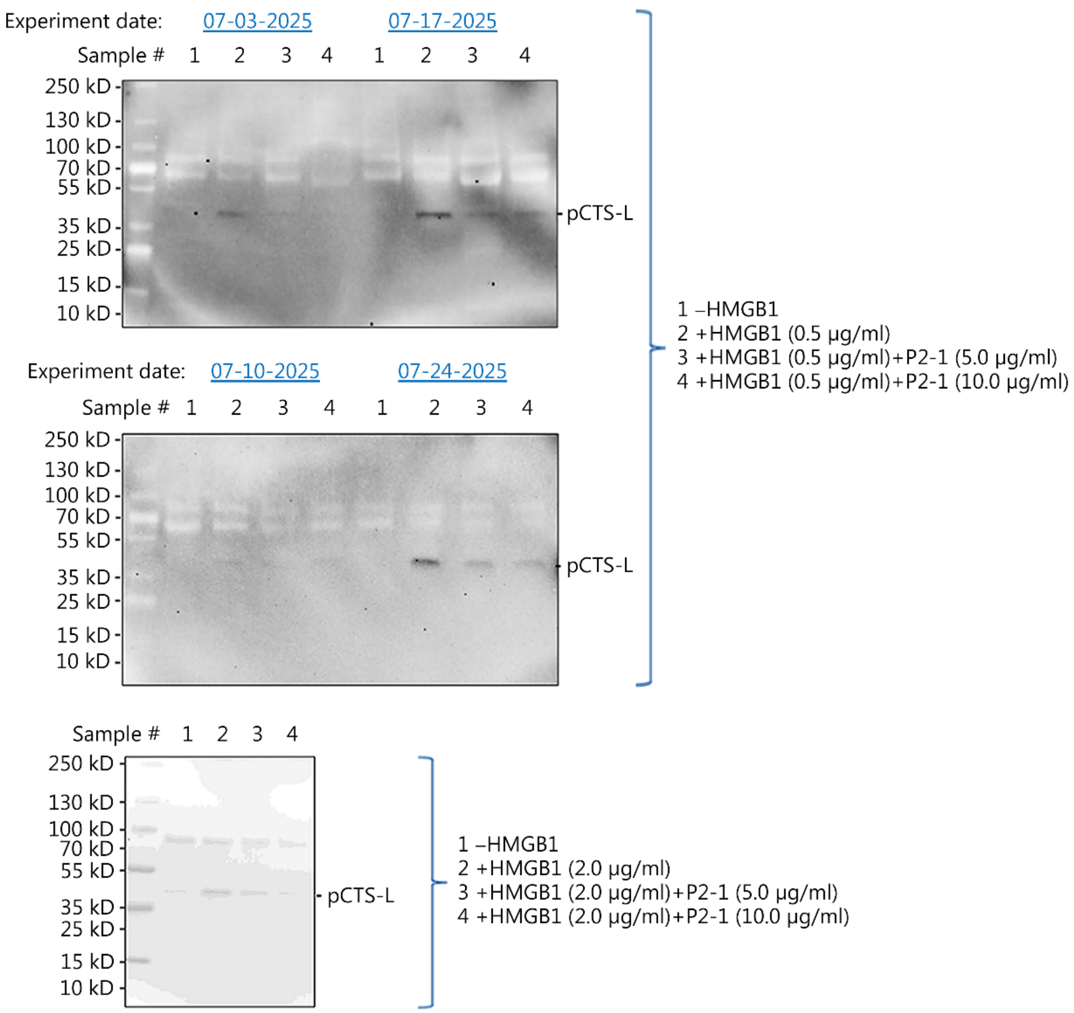


**Fig. S9** Full Western blotting analysis of the effect of P2-1 on HMGB1-induced pCTS-L release in human PBMCs. Human PBMCs were stimulated with HMGB1 (0.5 µg/ml) in the absence or presence of P2-1 (5.0 or 10.0 µg/ml) for 16 h, and the levels of pCTS-L in the cell-conditioned medium were determined by Western blotting, with sample loading normalized by volume of culture medium conditioned by an equivalent number of cells. Representative Western blotting images showing the dose-dependent inhibitory effect of P2-1 (5.0 and 10.0 µg/ml) on HMGB1-induced (0.5 µg/ml) pCTS-L secretion from human PBMCs. HMGB1 high mobility group box 1, pCTS-L procathepisn L, PBMCs PBMCs peripheral blood mononuclear cells

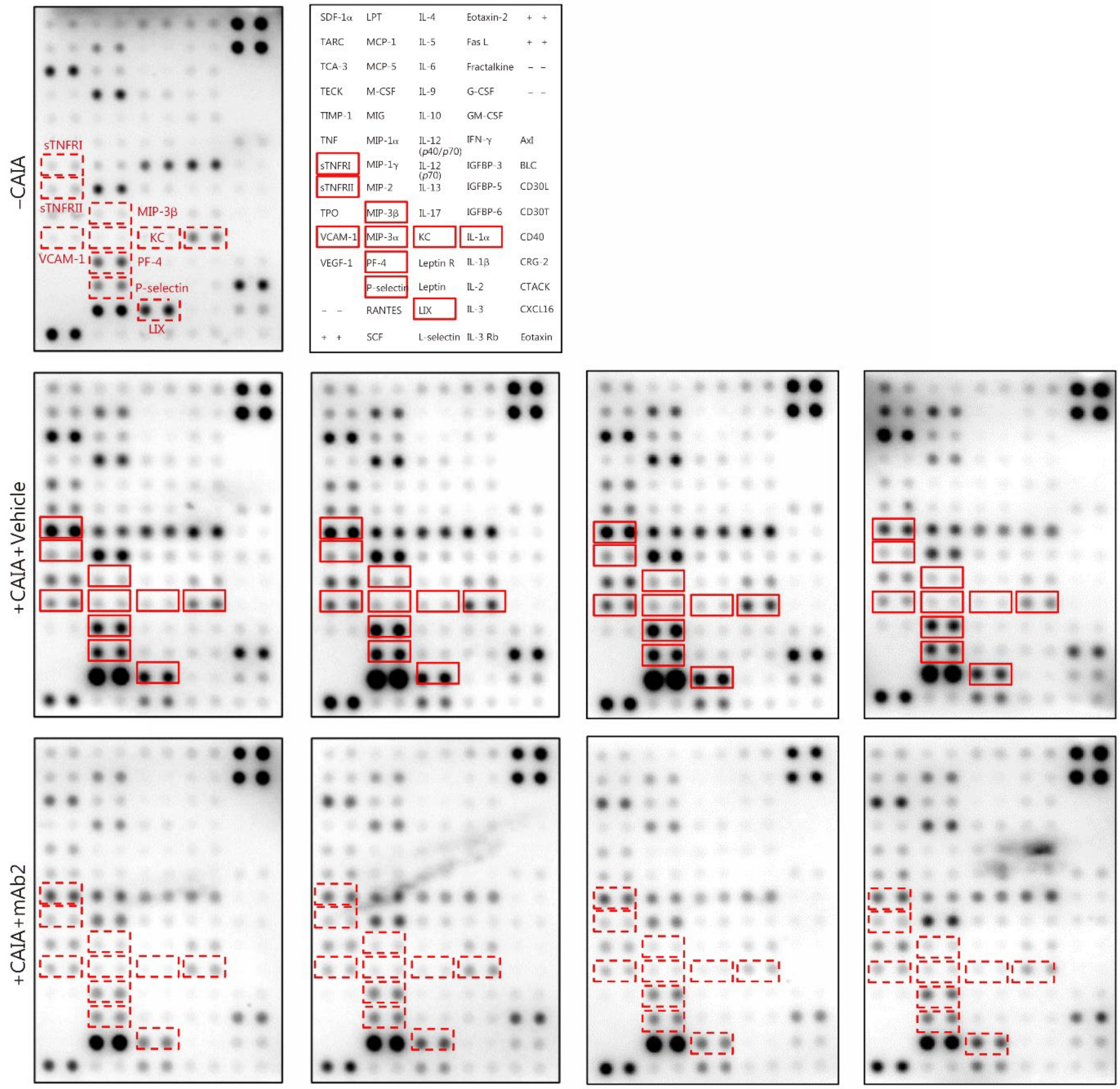


**Fig. S10** Representative cytokine antibody arrays illustrating the effect of mAb2 on collagen antibody-induced arthritis (CAIA)-induced joint inflammation. Joint soft tissue from BALB/c mice, subjected to CAIA and treated with a pCTS-L-neutralizing mAb2 (4.0 mg/kg, intraperitoneally on Days 6, 7, 8, and 9), was harvested on Day 10. The levels of various cytokines and chemokines were measured via cytokine antibody arrays. Representative images from cytokine antibody arrays of joint tissue lysates from a normal mouse ("- CAIA"), 4 vehicle-treated CAIA mice, and 4 pCTS-L-neutralizing mAb2-treated CAIA mice (harvested Day 10). These findings revealed visual reductions in various inflammatory cytokines and chemokines in the mAb2-treated samples. pCTS-L procathepsin L, IL interleukin, KC keratinocyte-

derived chemokine, LIX LPS-induced CXC chemokine, MIP macrophage inflammatory protein, PF-4 platelet factor 4, TNFR tumor necrosis factor receptor, VCAM vascular cell adhesion molecule

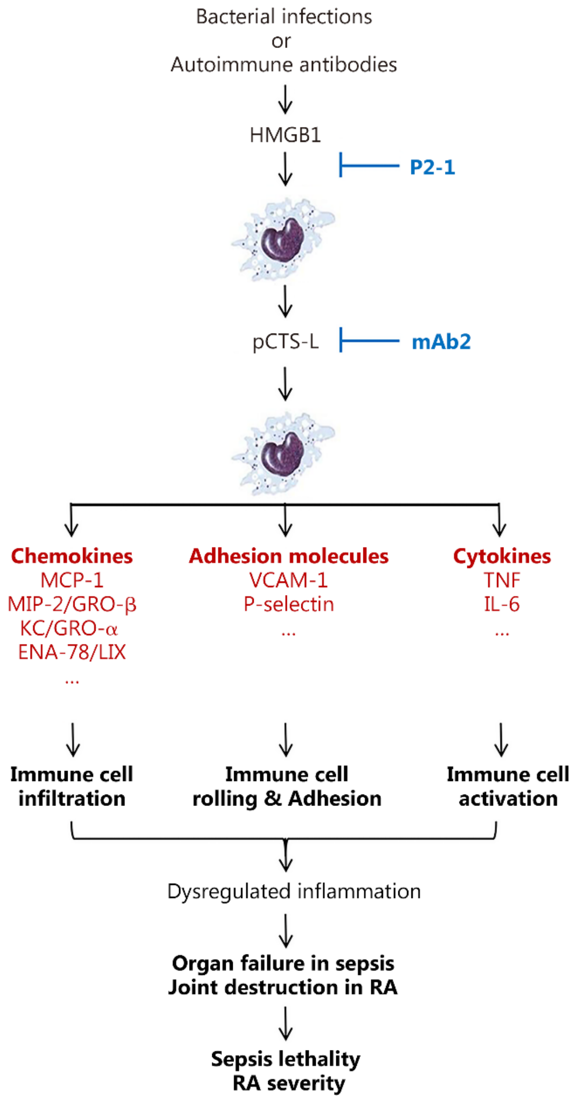


**Fig. S11** Proposed model for P2-1-mediated intervention in the inflammatory HMGB1-pCTS-L axis in sepsis and rheumatoid arthritis. Pathogenic inflammation in sepsis and rheumatoid arthritis (RA) is driven by a dysregulated innate immune response, involving complex interactions among chemokines, adhesion molecules, and proinflammatory cytokines. Chemokines (e.g., CCL20 and CXCL1) orchestrate immune cell infiltration to inflamed sites, whereas adhesion molecules (e.g., VCAM-1 and P-selectin) facilitate their extravasation. Key inflammatory mediators, including TNF, HMGB1, and pCTS-L, act through specific receptors (e.g., TNFRI/II, TLR4, and RAGE) to perpetuate a self-amplifying inflammatory cycle, ultimately causing organ failure in sepsis or joint destruction in RA. Our findings illustrate how P2-1, a peptide derived from the epitope of a detrimental anti-TN mAb9, directly binds HMGB1 and

competitively impairs the HMGB1-RAGE interaction, thereby selectively inhibiting HMGB1 uptake, macrophage pyroptosis, and HMGB1-induced *Ctsl* mRNA upregulation and pCTS-L secretion. This RAGE-specific interference would account for P2-1's precise inhibition of HMGB1-induced pCTS-L induction without broadly affecting other cytokines and chemokines. However, it remains unknown whether HMGB1 induces pCTS-L upregulation through RAGE or other off-target receptors. It will thus be important to determine whether P2-1's selective suppression of pCTS-L is abrogated by pharmacological or genetic blockade of RAGE or other off-target receptors, thereby establishing the receptor-specific mechanistic basis for P2-1's therapeutic precision. Given the critical role of pCTS-L in lethal sepsis [2] and its confirmed contribution to arthritis pathogenesis (as demonstrated herein), P2-1 has emerged as a targeted therapy that specifically disrupts the HMGB1-pCTS-L axis in inflammatory diseases. HMGB1 high mobility group box 1, RAGE receptor for advanced glycation end products, pCTS-L procathepsin L, mAb2 monoclonal antibody 2, TNFR tumor necrosis factor receptor, TLR Toll-like receptor, MCP monocyte chemoattractant protein, MIP macrophage inflammatory protein, GRO growth-regulated oncogene, KC keratinocyte-derived chemokine, ENA epithelial-derived neutrophil activating peptide, LIX LPS-induced CXC chemokine, VCAM vascular cell adhesion molecule, TNF tumor necrosis factor, IL interleukin, CCL chemokine (C-C motif) ligand, CXCL chemokine (C-X-C motif) ligand, Ctsl cathepsin L

**Table S1** Key reagent sources

| Reagent or resource | Source | Identifier |
|---|---|---|
| Antibodies | | |
| Mouse anti-β-actin antibody | Sigma-Aldrich, St. Louis, MO, USA | Cat. #A1978 |
| Mouse anti-murine procathepsin L (pCTS-L) monoclonal antibodies | Home-made | *Science Advances*, 2023 |
| Mouse anti-human tetranectin mAb8 and mAb9 | Home-made | *Science Translational Medicine*, 2000 |
| HRP-conjugated mouse IgG kappa-binding protein (m-IgGκ BP) | Santa Cruz Biotechnology, Inc., Dallas, TX, USA | Cat. #sc-516102 |
| Anti-Type II Collagen Antibody Cocktail (A2-10, D1-2G, D8-6, F10-21) | Chondrex Inc., Woodinville, WA, USA | Cat. #53100 |
| Chemicals | | |
| Human serum | Sigma-Aldrich, St. Louis, MO, USA | Cat. #H3667 |
| Recombinant human HMGB1 protein | Home-made | *Science*, 1999 |
| Dulbecco's modified Eagle medium (DMEM) | Invitrogen/Life Technologies, Carlsbad, CA, USA | Cat. #11995-065 |
| OPTI-MEM I reduced-serum medium | Thermo Fisher Scientific, Somerset, NI, USA | Cat. #31985062 |
| Penicillin/streptomycin | Invitrogen/Life Technologies, Carlsbad, CA, USA | Cat. #15140-122 |
| Trypan blue | Invitrogen/Life Technologies, Carlsbad, CA, USA | Cat. #15250-061 |
| Alexa Fluor 555 labeling kit | Thermo Fisher Scientific, Waltham, MA, USA | Cat. #A30007 |
| DAPI | Vector Laboratories, Inc., Newark, CA, USA | Cat. #H-1200 |
| Ketamine | Henry Schein Special Market, Moonachie, NJ, USA | Cat. #2480861 |
| Xylazine | Sigma-Aldrich, St. Louis, MO, USA | Cat. #X1251 |
| Critical commercial assays and SPR Sensor Chip | | |
| LDH Assay Kit | Pointe Scientific Inc. Canton, MI, USA | Cat. #L7572 |
| Murine cytokine antibody arrays | RayBiotech Inc., Peachtree Corners, GA, USA | Cat. #AAM-CYT-3-8 |

| Reagent or resource | Source | Identifier |
|---|---|---|
| Human cytokine antibody C3 arrays | RayBiotech Inc., Peachtree Corners, GA, USA | Cat. #AAH-CYT-3-8 |
| Nylon Von Frey filaments | Stoelting Co., Wood Dale, IL, USA | Cat. #58011 |
| High sensitivity carboxyl sensors | Nicoya Lifesciences, East Kitchener, ON, Canada | Cat. #SEN-HS-8-COOH |
| Experimental models: organisms/strains | | |
| BALB/c mice | Jackson Laboratory, Bar Harbor, ME, USA | Stock # 000651 |
| BALB/c mice | Charles River Laboratories, Wilmington, MA, USA | Strain Code: 194 |
| Software and algorithms | | |
| UN-SCAN-IT Gel analysis software version 7.1 | Silk Scientific Inc., Provo, UT, USA | - |

*mAb* monoclonal antibody, *HMGB1* high mobility group box 1

## References


1. Chen W, Qiang X, Wang Y, Zhu S, Li J, Babaev A, et al. Identification of tetranectin-targeting monoclonal antibodies to treat potentially lethal sepsis. Sci Transl Med. 2020;12(539):eaaz3833.
2. Zhu CS, Qiang X, Chen W, Li J, Lan X, Yang H, et al. Identification of procathepsin L (pCTS-L)-neutralizing monoclonal antibodies to treat potentially lethal sepsis. Sci Adv. 2023;9(5):eadf4313.
